# Impact of the Level of Adherence to Mediterranean Diet on the Parameters of Metabolic Syndrome: A Systematic Review and Meta-Analysis of Observational Studies

**DOI:** 10.3390/nu13051514

**Published:** 2021-04-30

**Authors:** Dimitra Rafailia Bakaloudi, Lydia Chrysoula, Evangelia Kotzakioulafi, Xenophon Theodoridis, Michail Chourdakis

**Affiliations:** Laboratory of Hygiene, Social & Preventive Medicine and Medical Statistics, School of Medicine, Faculty of Health Sciences, Aristotle University of Thessaloniki, 54124 Thessaloniki, Greece; dmpakalo@auth.gr (D.R.B.); chrysoulal@auth.gr (L.C.); evelinakotzak@hotmail.com (E.K.); xenophontheodoridis@gmail.com (X.T.)

**Keywords:** metabolic syndrome, Mediterranean diet adherence, Mediterranean dietary pattern

## Abstract

High adherence to the Mediterranean diet (MD) has been associated with a lower prevalence of Metabolic Syndrome (MetS). The present study aimed to investigate the impact of MD adherence on parameters of MetS. A systematic literature search was performed in PubMed, Cochrane Central Registry of Clinical Trials (CENTRAL), Scopus, EMBASE, Web of Science and Google Scholar databases. Observational studies that recorded adherence to MD and components/measures of the MetS, such as waist circumference (WC), blood pressure (BP), fasting blood glucose (FBG), high-density lipoprotein (HDL) cholesterol and triglycerides (TG), were included in this study. A total of 58 studies were included in our study. WC and TG were significantly lower in the high adherence MD group (SMD: −0.20, (95%CI: −0.40, −0.01), SMD: −0.27 (95%CI: −0.27, −0.11), respectively), while HDL cholesterol was significantly higher in the same group (SMD: −0.28 (95%CI: 0.07, 0.50). There was no difference in FBG and SBP among the two groups (SMD: −0.21 (95%CI: −0.54, 0.12) & SMD: −0.15 (95%CI: −0.38, 0.07), respectively). MD may have a positive impact on all parameters of MetS. However, further research is needed in this field.

## 1. Introduction

Metabolic Syndrome (MetS), also known as the syndrome X, belongs to the group of non-communicable diseases (NCDs) [1]. The prevalence of MetS has been closely related to socioeconomic factors, as well as lifestyle changes deriving from the impact of westernization on diet and health behavior [1]. Thereby, this transition has led to an increase in morbidity and mortality rates, forcing health systems to introduce more effective strategies so as to prevent the expansion of this epidemic [2]. According to the National Health and Nutrition Examination Survey (NHNES), the prevalence of MetS in US adults reached 34.2% during 2007–2012, with the highest rates observed in non-Hispanic white males and elderly >70 years of age [3]. A large analysis of cohort studies in European countries from 2000 to 2013 revealed that the prevalence of MetS ranged from 42.7%–78.2% for males and 24%–68.4% for females [4].

Μetabolic syndrome has been characterized by health professionals and scientists as a cluster of predefined metabolic conditions, namely, hyperglycemia, dyslipidemia, hypertension and central obesity [5]. Chronic low-grade inflammation is considered another important risk factor present in the pathogenesis of MetS [6]. Increased adipose tissue and circulation of inflammatory mediators triggered by excess intake of specific micronutrients comprise the two primary components, which induce proinflammatory responses [6]. Consequently, MetS has been linked to not only the development but also to the progression of other NCDs, such as cardiovascular disease (CVD), type 2 diabetes mellitus (T2DM), chronic respiratory diseases, etc. [7,8]. More specifically, it has been demonstrated that metabolic syndrome can increase the risk of CVD and mortality by 78% [9].

Currently, the most popular criteria used for the diagnosis of the MetS come from three different organizations, the World Health Organization (WHO) [10], the National Cholesterol Education Program in Adult Treatment Panel III (NCEP-ATP III), established slightly different criteria for the identification of MetS, excluding insulin resistance and using waist circumference, which are the most commonly applied criteria in clinical practice [11], and the International Diabetes Federation (IDF) that has also published similar definitions with regards to the MetS, however, diagnosis relies mainly on central obesity [12]. A summary of the diagnostic criteria of MetS can be found in Table 1.

Lifestyle modifications, focusing on dietary patterns and physical activity, may improve markers of MetS and further reduce the risk of development of NCDs [13]. Among various types of dietary treatments, there has been a great deal of evidence with regards to the potential benefits of the Mediterranean diet (MD) in the field of nutritional epidemiology [14]. The traditional MD can be characterized as a plant-based diet containing high amounts of monosaturated fats, omega-3 fatty acids, polyphenols, vitamins and antioxidants, and low amounts of saturated fats and ethanol. With respect to nutrient content, the MD provides approximately 35%–45% fats (of which about 20% derives from monounsaturated fatty acids (MUFAs), 5% from polyunsaturated fatty acids (PUFAs) and 9% from saturated fatty acids (SFAs)), 15% protein and 45% carbohydrates [15]. However, what makes the MD distinct from other dietary patterns is the presence of various food components, including unrefined cereals, legumes, fish, vegetables, fruit, nuts, moderate amounts of wine and, most importantly, olive oil, which is considered the traditional symbol of MD [16].

Over the years, different dietary index scores have been developed for assessing the degree of adherence to the MD [17]. These composite scores aim to measure overall dietary quality with the use of validated food frequency questionnaires (FFQs) [17,18]. Data obtained from FFQs are combined within specific groups, food combinations or nutrients found typically in the MD, in which a specific value is assigned based on a predefined calculation [19]. Ratings resulting from MD scores (MDSs) from all groups are often categorized as low, moderate or high, reflecting the adherence level to MD for each subject [17,18]. As there is no specific rule or consensus as to how the adherence level of different MDSs should be interpreted, low scores indicate poor adherence, whereas higher scores indicate good adherence to MD or otherwise described by the authors. In general, high adherence is the result of frequent consumption in adequate quantities of beneficial components, such as fruits, vegetables, legumes, fish, nuts, whole grain products and olive oil, whereas there is a low intake of alcohol, meat and SFA [20,21].

Several studies have revealed an inverse association between adherence to MD and risk of obesity, CVDs, T2DM as well as all-cause mortality [22,23,24,25,26,27]. The potential advantages relate to the synergic effect and mechanisms of specific nutrients that have a direct impact on all risk markers of MetS, namely, WC, HDL, TG, FBG, BP, as well as systemic inflammation [28]. Even though the positive impact of MD on risk and occurrence of MetS has been previously confirmed [29,30], there have not been any analyses evaluating how different levels of adherence to MD could favorably impact each parameter of MetS.

Therefore, the purpose of this study was to examine the impact of low and high adherence to MD on the parameters of MetS.

## 2. Materials and Methods

This study is a systematic review and a meta-analysis which was conducted according to the Meta-analyses Of Observational Studies in Epidemiology (MOOSE) statement (Supplementary File S1). The protocol of this systematic review and meta-analysis was submitted in the OSF platform (https://osf.io/n4ja8/ accessed on 5 March 2021).

### 2.1. Literature Search

A systematic literature search was conducted in the following electronic databases PubMed, EMBASE, Google Scholar, Scopus, Web of Science and Cochrane Central Registry of Clinical Trials (until 11 January 2021) in all fields option using the following search string: (“Mediterranean diet”) AND (Adherence) for the PubMed database, which was modified accordingly for the other search engines (search terms and keywords of our search strategy can be found in Supplementary File S2). Additional relevant studies were searched by references screening of the articles retrieved.

### 2.2. Study Selection-Eligibility Criteria

Eligible studies for inclusion to systematic review were original observational studies that investigated the impact of MD adherence on three or more parameters of MetS (WC, HDL, TG, SBP and FBG), according to the revised criteria NCEP ATP III [11], in the adult population, using a validated tool or scoring algorithm. MDSs developed by Panagiotakos et al. [31], Sofi et al. [32] and Trichopoulou et al. [21], as well as the PREDIMED MD Adherence Screener (MEDAS) score [33], the short MDS produced by Martinez Gonzalez et al. [34] the serving MDS [35], the Mediterranean-Style Dietary Pattern Score (MSDPS) by Rumawas et al. [36], the MD quality index [37], the relative MD system [38], and modified versions of MDSs [39,40,41,42,43,44,45,46,47,48,49], were used in our included studies. A summary of the diagnostic criteria of MetS can be found in Table 1. Studies that were not published as original papers (e.g., abstracts, conference papers, editorials and commentaries, etc.) were excluded. Additionally, manuscripts that did not provide adequate data regarding low and high adherence to MD were also excluded from this analysis. Only studies in English and Spanish language were part of our review.

### 2.3. Data Extraction

Records of our search results were imported into a reference management software (Endnote X9 for windows-by Clarivate Analytics USA) and two reviewers (LC, DB), after the removal of duplicates, assessed the studies for eligibility. Any disagreements were solved by a third reviewer (EK). Data extraction was performed independently by the above-mentioned two reviewers using a pre-specified standardized Microsoft^®^ excel form and was checked for accuracy by a third reviewer (EK). In cases of missing data, corresponding authors were contacted by email in order to retrieve any additional data.

The primary outcome of our study was to investigate the impact of high adherence to MD compared to low adherence to MD on the five parameters of MetS according to the NCEP ATP III [11] revised criteria for diagnosis.

### 2.4. Quality Assessment of Included Studies

The quality of the eligible studies was assessed using the Newcastle Ottawa Scale (NOS) adjusted version for cross-sectional studies by two independent authors (LC and DB) [50]. Any disagreements that arose were solved by consensus and by the involvement of a third author (EK). Sensitivity analysis was further performed after the exclusion of low-quality studies (NOS < 7).

### 2.5. Statistical Analysis

Means and standard deviations (SD) from eligible studies reported high and low MD adherence for each parameter of MetS were used. Wherever it was necessary, and data were presented as median, minimum or maximum values or 95% confidence intervals (CI), conversion to mean and SD was performed [51,52,53,54]. When values of FBG, TG and HDL cholesterol were presented as mmol/L, conversion to mg/dL was employed using the Omni calculator [55]. The inverse variance method was used in order to estimate the weight of each study. The random effects model was used due to higher methodological heterogeneity among the included studies [56,57]. Moreover, Hedge’s g was used as effect size and standardized mean difference (SMD) as a summary statistic model due to the heterogenous scores using in included studies for the definition of low and high adherence to MD [56]. Estimation of heterogeneity was performed with Cochrane Q test (*p* < 0.1: existence of heterogeneity) and I^2^ statistic [56,57]. I^2^ values >50% indicated substantial heterogeneity across studies. Publication bias was assessed with funnel plots and Egger’s test [53]. All statistical analyses were performed using the R software developed at Bell Laboratories (formerly AT&T, now Lucent Technologies version 4.0.2).

## 3. Results

### 3.1. Search Results

**Figure 1:**
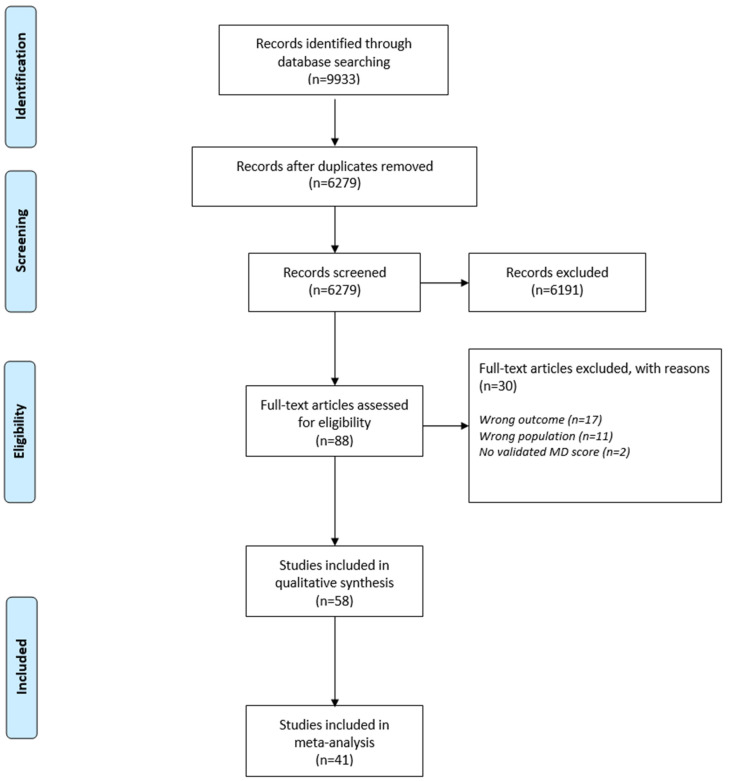
Flow diagram of the eligibility process of included studies.

A total of 9933 studies were identified through the literature search. After removing 3654 duplicates, 6279 studies were detailed screened for eligibility. The process of eligibility of our included studies can be found in the flow diagram in Figure 1. Not relevant to the topic examined studies, studies including population <18 years old, studies in which validated tool for assessment of MD were not used and in which the level of adherence was not clearly described were excluded. Overall, 58 studies were characterized as acceptable for the systematic review [39,40,43,44,45,46,47,49,58,59,60,61,62,63,64,65,66,67,68,69,70,71,72,73,74,75,76,77,78,79,80,81,82,83,84,85,86,87,88,89,90,91,92,93,94,95,96,97,98,99,100,101,102,103,104,105,106,107] and 41 for the meta-analysis [45,46,47,49,58,59,60,61,62,63,64,65,66,67,68,69,70,71,72,73,74,75,76,77,78,79,80,81,82,83,84,85,86,87,88,90,91,92,93,94]. Authors of studies in which data were not adequate for our systematic review or/and meta-analysis were contacted by email requesting supplemental data without any response received.

### 3.2. Quality Assessment

The quality of the 58 included studies was examined according to the NOS [50]. Five studies were characterized as unsatisfactory due to their ratings (2–4 stars) [43,67,75,80,107], whereas for 17 studies the quality was only satisfactory (5–6 stars) [39,49,61,63,71,86,92,93,94,96,97,100,104,105]. The majority of the included studies (*n* = 28) [40,44,45,47,58,59,60,62,65,68,69,70,72,74,76,77,79,81,82,83,84,85,87,88,95,101,103,106] were good quality studies (7–8 stars), and eight studies were at the top of quality studies scoring 9 stars [46,64,66,73,91,98,99,102]. More information regarding the assessment of quality according to the NOS can be found in Supplementary File S3.

### 3.3. Publication Bias

Funnel plots of studies included in our meta-analysis regarding each parameter of MetS can be found in Supplementary Figure S1a–e. Both the symmetry of funnel plots and Egger’s test results confirm the absence of publication bias in all parameters of MetS except TG. Eggers’s test results were *p* = 0.8325 referred to WC, *p* = 0.2177 referred to HDL, *p* = 0.04598 referred to TG, *p* = 0.8533 referred to SBP, and *p* = 0.4677 referred to FGL.

### 3.4. Study Characteristics

Characteristics of the included studies can be found in Table 2 for studies included in the systematic review and Table 3 for studies included in the meta-analysis, in which the country origin, the number, the mean age as well as the specific group of participants, and the MD assessment tool are included. In total, 74,058 adult subjects from all over the world (Australia, Chile, Finland, France, Greece, Iran, Italy, Korea, Morocco, The Netherlands, Poland, Spain, Sweden, Taiwan, Turkey, UK and USA) who followed an MD were examined.

### 3.5. Result on Components of MetS

#### 3.5.1. Waist Circumference (WC)

In three studies in which OR of the prevalence of WC >102 cm for males and >88 cm for females was used as a measure of the effect, low odds for this outcome were observed in the groups of high adherence to MD [39,99,104]. Moreover, in the study by Mirmiran et al. [103], in which the incidence of abnormalities during 3 years follow-up was examined and expressed as OR, a lower incidence was found in the high adherence group, but this was not significant (*p* > 0.05). In Aridi et al. [95] and Mattei et al. [101], a significantly lower mean WC was found in the high adherence groups, as well as in 3 more studies [98,102,107] in which follow-up results were obtained. In Rumavas et al. [106], a significantly lower geometric mean of WC in the high adherence group was reported (*p* < 0.001), and in Steffen et al., the prevalence of subjects reporting an unhealthy WC was significantly lower in the high adherence group [44]. Only in one study, WC did not differ between the low and the high adherence group [40].

**Figure 2:**
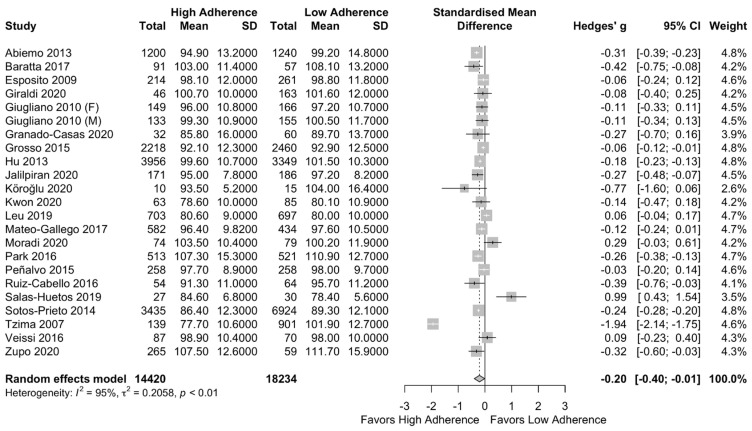
Forest plot of the impact of level of adherence to MD on WC (cm).

The meta-analysis results showed a lower WC in the low adherence group [SMD: −0.20, (95%CI: −0.40, −0.01)] with a high heterogeneity among studies (I^2^ = 95%) as presented in Figure 2. In order to explore the heterogeneity, a subgroup analysis of higher quality (NOS > 7) and lower quality (NOS < 7) studies was performed, which led to not significant results (SMD: −0.19 (95%CI: −0.48, 0.10)) and I^2^ = 96% as can be seen in Supplementary Figure S2.

#### 3.5.2. HDL Cholesterol

In subjects reporting high adherence to MD, the ORs of HDL cholesterol <40 mg/dL for males and <50 mg/dL for females were lower, compared to low adherers but not significantly [39,99,104], even after three years of follow-up [103]. Mean and geometric mean HDL cholesterol concentrations were increased in the high adherence groups [40,97,98,100,105,106,107]. A significantly increased (*p* = 0.0258) HDL cholesterol concentration in the high adherence group was reported by Yang et al. [43]. In Aridi et al. [95] and Steffen et al. [44], the percentage of subjects with increased HDL cholesterol was higher in the high MD adherence group compared to the low adherence group. On the contrary, in two studies, the mean HDL cholesterol concentration was higher in low adherence compared to high adherence groups [101,102]. Only in Barnaba et al., no difference regarding the mean HDL concentration was found between the moderate-high adherence group and the low adherence to MD group [96].

**Figure 3:**
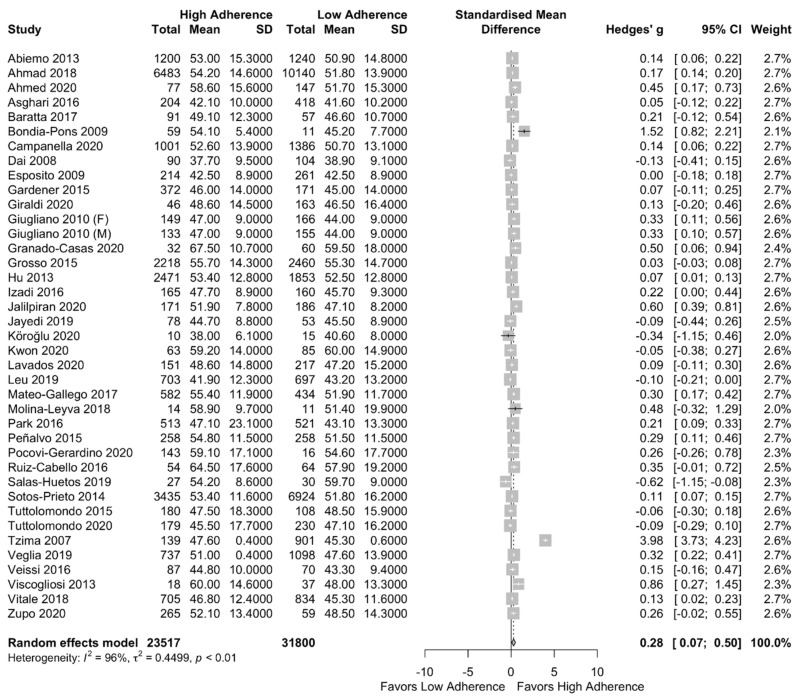
Forest plot of the impact of level of adherence to the MD on HDL cholesterol (mg/dL).

Results of our meta-analysis can be found in the forest plot of Figure 3. Significant higher HDL cholesterol concentration in the high adherence to MD group was observed (SMD: 0.28 (95%CI: 0.07, 0.50)) with high heterogeneity among the included studies I^2^ = 96%.

In the subgroup analysis (based on the quality of studies per NOS), the significantly increased HDL cholesterol concentration was remained after excluding the low-quality studies (SMD: 0.36 (95% CI: 0.03, 0.68)) with I^2^ = 98% as can be seen in Supplementary Figure S3.

#### 3.5.3. Serum Triglycerides

Regarding the studies which used OR as a measure of effect, in three studies [99,103,104], the ORs of having TG concentration above 150 mg/dL were lower for the high adherence group, and in only one study, the OR was higher [39]. Means and geometric means TG concentration were observed to be lower in high adherence groups [40,43,98,100,102,105,106,107] compared to the low adherence groups. Similarly, in Steffen et al. [44], a significantly lower percentage was reported for increased TG concentration in the high adherence to MD group compared to the low adherence group. In contrast, in two studies led by Barnaba and by Matei, a higher concentration of TG was reported in the high-moderate adherence group and in the high adherence group, respectively, compared to the low adherence group [96,101]. Additionally, in the study led by Aridi, a higher, but not significant, percentage reported increased TG concentration in the high adherence to MD group compared to the low adherence group [95].

**Figure 4:**
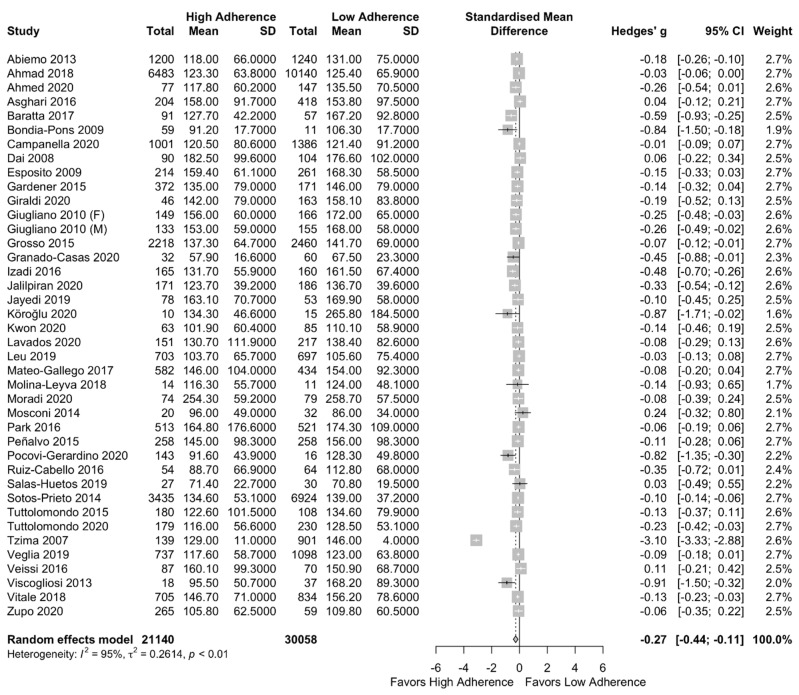
Forest plot of the impact of level of adherence to the MD on serum TG (mg/dL).

After performing the meta-analysis, TG concentration was found to be lower in the high adherence to MD group compared to the low adherence group (SMD: −0.27 (95%CI: −0.44, −0.11)) with a high heterogeneity among the studies I^2^ = 95% as is presented in Figure 4. In the subgroup analysis of low- and high-quality studies, the same results also remained after excluding the low-quality studies (SMD: −0.29 (95% CI: −0.52, −0.05)) with I^2^ = 97% (Supplementary Figure S4).

#### 3.5.4. Fasting Blood Glucose

In 2 studies by Alvarez-Leon et al. [39] and Mirmiran et al. [103], ORs of having FBG >180 mg/dL were higher in the high adherence group to MD in comparison to the low adherence group, whereas in 2 other studies were opposite (ORs were lower regarding in the high adherence group) [99,104]. Means and geometric means concentration of FBG were lower in high adherers compared to low MD adherers [43,97,98,100,105,106]. According to Aridi et al. and Steffen et al. studies, a lower percentage of subjects presented FBG concentration >110 mg/dL in the high adherence group compared to the low adherence to MD group [44,95]. However, the mean concentration of FBG was increased in high adherers compared to low adherers [40,102,107] and low-moderate adherers [96].

**Figure 5:**
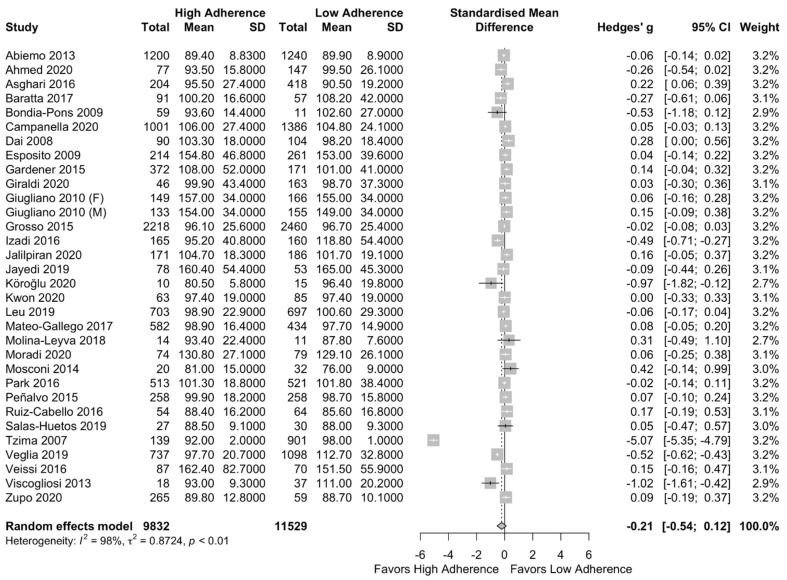
Forest plot of the impact of level of adherence to MD on FBG (mg/dL).

The meta-analysis results can be found in Figure 5. There was no difference in FBG between the two groups (SMD: −0.21 (95%CI: −0.54, 0.12)). The above did not change after performing a subgroup analysis per the NOS classification (SMD: −0.24 (95%CI: −0.70, 0.22) for the high-quality studies) as can be seen in Supplementary Figure S5.

#### 3.5.5. Systolic Blood Pressure (SBP)

Regarding the SBP, in four studies, the ORs of a measuring SBP >130 mmHg were lower in subjects reporting high adherence to MD compared to low adherers [39,99,103,104]. Moreover, means and geometric means of SBP were lower in the high adherence group compared to the low adherence group [40,98,102,106]. According to Aridi et al. [95] and Steffen et al. [44], lower percentages of subjects presented SBP >130 mmHg from the high adherence to MD group compared to the low adherence group. Three studies reported the opposite (higher SBP was observed in higher adherence to MD) [43,101,107].

**Figure 6:**
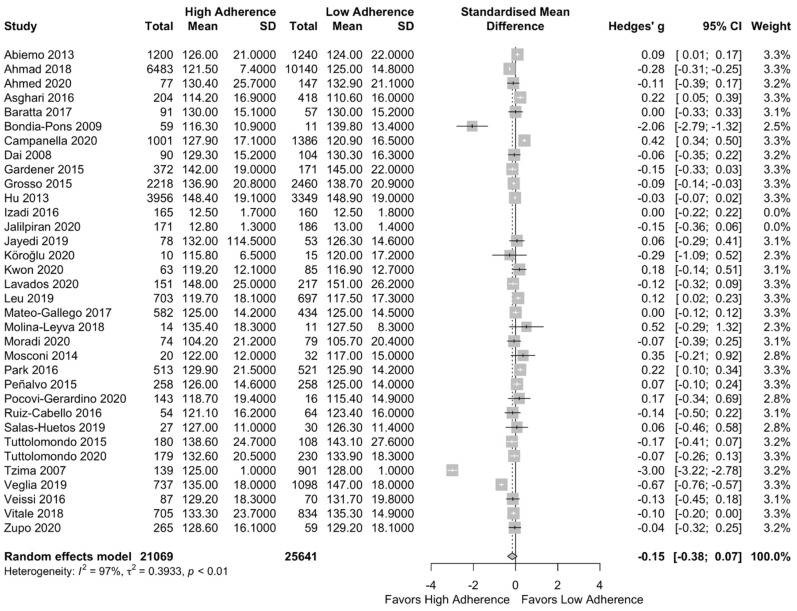
Forest plot of the impact of level of adherence to the MD on SBP (mg/dL)—*n* = 25,641.

Meta-analysis results can be found in Figure 6. Lower SBP was observed in the high adherence group but not significant (SMD:−0.15 (95% CI: −0.38, 0.07)) with high heterogeneity across the included studies (I^2^ = 97%). This result did not change after the performance of a subgroup analysis based on the quality of studies (SMD: −0.25 (95%CI: −0.60, 0.10), I^2^ = 98%) as can be seen in Supplementary Figure S6.

## 4. Discussion

Our systematic review and meta-analysis aimed to investigate the association between a low and high level of adherence to MD and risk parameters of MetS, according to the NCEP-ATP III criteria. The present study, examining 41 observational studies, revealed a positive impact of MD on the five components of MetS, including WC, HDL, TG, FG and BP. Although a previous meta-analysis conducted by Kastorini et al. [30] explored the effect of MD on MetS prevalence, including its components, this is the first meta-analysis estimating the impact of the level of adherence to MD on each parameter of MetS according to evidence obtained by MD adherence scores.

With regards to abdominal obesity, our results showed a significant inverse association between WC and adherence to MD. Only one study [40] did not find any statistical difference in WC between the different levels of adherence to MD groups, which could be attributed to the underlying health condition of participants (CKD patients). Increased WC, which was detected in the low adherence to MD subjects, along with the accumulation of visceral fat, have been linked to the presence of low-grade systemic inflammation, increased oxidative stress and overexpression of pro-inflammatory cytokines, including CRP, IL-6 and TNF-a [109,110]. These metabolic abnormalities have a direct impact on other biochemical risk markers of MetS, and more specifically HDL, TG and FG, which consequently stimulate atherogenesis and mediate insulin resistance [111]. The high content of antioxidants, polyphenols and fiber found in MD have been previously associated with decreased systemic inflammation and central obesity, which could explain its beneficial effect [112,113]. Moreover, an enhanced with nuts MD was found to be helpful regarding the maintenance of body weight status [114,115].

A significantly positive correlation was also found between high adherence to MD and HDL cholesterol concentration. Our findings are consistent with previously reported data from randomized controlled trials (RCTs), in which a Mediterranean dietary pattern improved HDL cholesterol concentration and the overall lipid profile [116,117,118]. Increased intake of olive oil, polyphenols, antioxidants as well as an optimal ratio of MUFA:SFA, through the adherence to MD, seemed to have a synergistic effect on various mechanisms of lipid metabolism by promoting changes on the overall composition of HDL cholesterol particles, increased antioxidant and cholesterol efflux capacity [117,119]. Furthermore, a higher HDL concentration observed in high MD adherers could potentially be a secondary effect closely related to lower mean values of central obesity, as aforementioned, and improved cardiometabolic risk markers.

According to our results, an inverse significant association was observed between TGs concentration and adherence to MD. In a large network meta-analysis performed by Tsartsou et al. [108], the protective effect of MD on the overall lipid profile, including TGs, was also demonstrated. These findings were mainly attributed to the high content of olive oil polyphenols and oleic acid as part of the MD [108]. Another meta-analysis of RCTs, investigating the effect of plant oils on blood lipids, had also reported a decrease in TG concentration from the use of diets rich in olive oil [120]. Notwithstanding, it was demonstrated that oils rich in omega-3-fatty acids (n-3 FAs) caused a greater decrease in TGs than olive oil [120]. The metabolic mechanisms responsible for these changes are related to the types of fatty acids, i.e., MUFAS and n-3 FAs, which have the ability to suppress postprandial TGs, enhance TG clearance, decrease the activity of TG lipase and the overall TG synthesis [121,122,123].

Taking the above into consideration, where the mean values of WC, HDL cholesterol and serum TG concentration were significantly closer to normal in the high adherence to MD groups compared to the low adherence group, we conclude that the level of adherence to MD could play an important role to ameliorate the obesity level and the impaired lipid profile, in combination or not with appropriate pharmacological treatment.

With respect to FBG, an inverse correlation was demonstrated between MD levels of adherence and FBG, which, however, was not statistically significant. A possible explanation for that could be the high number of individuals diagnosed with diabetes or at diabetic risk who participated in the studies [49,61,65,68,69,70,71,74,81,92], along with other confounding factors (e.g., age, BMI, medication, etc.). However, the fact that mean values of FBG in both high and low adherers were within the normal range led us to the conclusion that MD adherence can have a positive impact on glycemic control regardless of the level of adherence. Sufficient evidence exists supporting the positive effect of adherence to MD so as to improve glycemic control and decrease the overall risk of T2DM [124]. A systematic review of 17 studies assessing the effect of MD on the incidence of T2DM revealed that high adherence to MD was significantly correlated with improved FBG concentration and HbA1c in diabetic patients [125]. Additionally, both RCTs and prospective cohort studies have also confirmed the benefits of MD on glycemic control over other diets among different subgroups of the population, including healthy individuals, individuals with high CVD/T2DM risk or diabetic patients [65,126,127]. These outcomes have been closely related to the composition of MD, which is rich in anti-inflammatory compounds, as well as to its enhanced activity of glucagon-like peptide (GLP-1) hormone and to changes in gut microbiome caused by MD [48]. Notwithstanding, a meta-analysis by Ajala et al. on 20 RCTs demonstrated that not only MD but also low-carbohydrate, low-glycemic-index and high protein diets could enhance the cardiometabolic profile [128].

Regarding SBP and adherence to MD level, we have also found an inverse but non-statistically significant association. Hypertension is considered a major risk factor for endothelial dysfunction and the development of CVDs [129]. It has been previously demonstrated that prolonged adherence to MD can decrease both SBP and DBP [130].

According to our included studies, in a vast majority, the mean SBP was <130 mmHg in both low and high adherence to MD groups. Consequently, we can conclude that even a poor adherence to MD can positively influence SBP. This conclusion is in accordance with existing data from previously published studies that have reported a significant inverse correlation between adherence of MD and BP [131,132]. Moreover, two recent meta-analyses showed that MD could significantly reduce BP when compared to control diets [133,134]. In addition, a greater decrease in BP was recorded for subjects presented with higher BP at baseline and in studies with a longer duration of the intervention [133]. Various nutrients included in MD exerted beneficial effects through improved vasodilation and endothelial function such as nitric oxides, flavonoids and minerals [135].

The benefits of MD adherence are not limited to the five parameters of MetS [136]. MiRNAs were found to be better regulated in obese patients following an MD [137]. Recent studies have shown that an MD reduces serum inflammatory markers as well as the incidence of stroke, CVD and breast cancer [138,139]. Moreover, MD was recommended as a diet that can help women with menopause-related symptoms and needs [140].

Our study can be characterized by several strengths. According to our knowledge, this is the first systematic review and meta-analysis that aimed to examine the impact of the level of adherence to an MD on the parameters of MetS. Moreover, the great number of the studies included and the subjects examined (*n* = 74,058), whose origin covered a significant part of the world, made our results quite representative. Furthermore, publication biases were not detected in our study, except from the studies included for the TG parameter in which the *p*-value of Egger’s test was not rounded up 0.04598. In addition, the fact that we have included studies that used validated MD adherence scores in order to assess the level of adherence to MD increased the accuracy of our conclusions. The limitations of our study mainly concerned the heterogeneity in the included studies. High heterogeneity was detected for all parameters of MetS, which was potentially due to the different types of population (i.e., ethnicity) and health status (i.e., healthy, obese/overweight and diagnosed conditions) across all included studies, as well as to the difference between sample sizes and the use of a variety of MDS. The presence of high heterogeneity in population samples and the fact that subjects under pharmacological treatment were not excluded do not allow for inference of our results regarding the role of MD. Over and above, the variety of MDSs used to assess adherence among studies introduces biases due to the different ways of classification and quantification of food components. Furthermore, levels of adherence to MD may be perceived differently, depending on the geographical location and, thus, produce additional bias. For example, high adherers living in Mediterranean regions might have a greater intake of specific foods when compared to high adherers residing in non-Mediterranean regions. Moreover, the conversion of data whenever necessary for unification of the quantitative analysis adds to our study’s limitations. Moreover, we have included studies published in English and Spanish; therefore, studies published in a different language were not a part of this study.

## 5. Conclusions

High adherence to MD can have a positive impact on all parameters of MetS. In addition, there is sufficient evidence suggesting that long-term consumption of MD can protect from obesity and improve cardiometabolic risk markers, including the markers used for the diagnosis of MetS. Although high heterogeneity was identified across the included studies, our results support previous findings and point to the potential biases that may derive from the use of MDSs. Furthermore, it remains still unclear whether MD exerts the same beneficial effect on both unhealthy and healthy populations; therefore, further research is needed in this field.

## Figures and Tables

**Table 1:** Published definitions and criteria for the diagnoses of MetS by the WHO, NCEP-ATP III and IDF.

Organization	Criteria
WHO (1998) [10]	Impaired glucose intolerance or diabetes and insulin resistance *Two or more of the following risk markers:* BP ≥ 160/90 mmHg Serum TG concentration >150 mg/dL HDL cholesterol concentration <35 mg/dL (males) and <39 mg/dL (females) Abdominal obesity: waist to hip ratio >0.90 (males) and >0.85 (females) and/or BMI > 30 kg/m^2^ Microalbuminuria ≥ 20 μg/min
NCEP-ATP III (2002) [11]	*Three or more of the following risk markers:* Abdominal obesity: WC > 102 cm (males) and >88 cm (females) Serum TG ≥ 150 mg/dL HDL cholesterol <40 mg/dL (males) and <50 mg/dL (females) BP ≥ 130/85 mmHg FΒG ≥ 110 mg/dL
IDF (2006) [12]	Central adiposity ^a^ *Plus two or more of the following markers* FBG > 100 mg/dL or diagnosed diabetes HDL cholesterol <40 mg/dL (males) and <50 mg/dL (females) or treatment for low HDL concentration Serum TG > 150 mg/dL or treatment for hypertriglyceridemia BP > 130/85 mmHg or treatment for hypertension

WHO: World Health Organization, NCEP-ATP III: National Cholesterol Education Program in Adult Treatment Panel III, IDF: International Diabetes Federation, HDL: High-Density Lipoprotein, TG: Triglycerides and FBG: Fasting Blood Glucose. ^a^ Ethnic-specific WC values: Europe ≥94 cm for males and ≥80 cm for females; South Asia and China ≥90 cm for males and ≥80 cm for females; Japan ≥85 cm for males and ≥90 cm for females.

**Table 2:** Characteristics of studies included only in the systematic review.

Study ID (Country)	No of Participants (F/M)	Mean Age (Years)	Population	MD Assessment Tool	WC (cm)	HDL Cholesterol (mg/dL)	TG (mg/dL)	FBG (mg/dL)	SBP (mmHg)	Measure of Effect
Alvarez-Leon 2006 (Canary Islands) [39]	578 (329/249)	≥18 ^1^	General population	Semi-quant FFQ 81 to calculate Specific food item score (10-item) [39]	L = 1 H = 0.77 [0.38–1.56]	L = 1 H = 0.90 [0.56–1.42]	L = 1 H = 1.05 [0.63–1.75]	L = 1 H = 2.46 [1.13–5.37] *	L = 1 H = 0.58 [0.34–0.99] *	OR [95%CI]
Aridi 2020 (Australia) [95]	3245 (1753/1492)	48.6 (17.6)	General population	Trichopoulou MDS [21]	L = 94.5 (14.7) H = 90.7 (13.3) *	L = 88.7% H= 89.9%	L = 83.1% H = 85.8%	L = 6.1% H = 5.7%	L = 123.6 (18.8) H = 122.1 (18.4)	Mean (SD)/ %Prevalence
Barnaba 2020 (Italy) [96]	349 (228/121)	18–86 ^1^	General population	MD serving score [35]	No info	L + M = 52.2 (11.1) H = 52.2 (13.4)	L + M = 107.5 (54.4) H = 110 (43.42)	L + M = 98.1 (12.2) H = 103.5 (11.76)	No info	Mean (SD)
Huang 2013 (Sweden) [40]	187 (0/187)	70	Elderly population with CKD	Modified Trichopoulou MDS 14-item [21]	L = 97 (10) H = 97 (11)	L = 47 (14) H = 48 (14)	L = 127.8 (59.9) H = 122.2 (70.8)	L = 103 (20) H = 106 (28)	L = 149 (19) H = 148 (19)	Mean (SD)
Karayiannis 2017 (Greece) [97]	142 (0/142)	37.8 (5.4)	Subjects without systemic diseases, cryptorchidism or varicocele, microorchidism, vasectomy or hormonal treatment in the last six months	MDS by Panagiotakos 0–55 points [31]	No info	L = 49.4 (11.3) H = 50.4 (10.6)	L = 107.9 (39.3) H = 84.3 (27.1)	L = 89.6 (9.1) H = 86.4 (8.3)	No info	Mean (SD)
Kesse-Geyot 2013 (France) [98]	1881 (668/1213)	49.7 (6.2)	General population	Trichopoulou MDS—9 points [21]	L = 84.21 (0.9) H = 82.8 (0.96)	L = 58 (1.19) H = 58.8 (1.2)	L = 88.5 (35.4) H = 84.07 (2.65)	L = 90.7 (0.4) H = 90.4 (0.7)	L = 128.7 (1.4) H = 127.67 (1.42)	Mean (SD)
Kim 2018 (Korea) [99]	2349 (1159/1190)	19–65 ^1^	General population	Modified MDS -9 points [41]	L = 1 H = 0.45 [0.31–0.66] *	L = 1 H = 0.89 [0.70–1.13] *	L = 1 H = 0.72 [0.55–0.94] *	L = 1 H = 0.83 [0.63–1.10] *	L = 1 H = 0.99 [0.74–1.34] *	OR
Mahdavi-Roshnan 2017 (Iran) [100]	344 (154/190)	L = 59.0 (8.30) H = 58.0 (9.36)	Subjects with CVD risk factors	PREDIMED MEDAS score -14 points [33]	No info	L = 42.81 (8.34) H = 43.3 (8.23)	L = 209.61 (399.33) H = 155.83 (87.63)	L = 116.4 (66.9) H = 105.9 (66.1)	No info	OR/ Mean (SD)
Mattei 2017 (US) [101]	1194 (No info)	L = 56.6 (7.9) H = 57.2 (7.7)	Subjects with no severe health conditions or cognitive impairments	Trichopoulou MDS—9 points [21]	L = 103 (14) H = 102 (13) *	L = 46.3 (12.5) H = 45.96 (12.3)	L = 163 (93) H = 165 (127)	L = 115 (53) H = 112 (36) *	L = 135 (19) H = 137 (20)	Mean (SD)
Mayr 2019 (Australia) [102]	37 (No info)	No info	Patients with coronary heart disease	PREDIMED MEDAS score 14—item [33]	L = 103.5 (3.4) H = 100.7 (3.3) *	L = 48.7 (6.5) H = 46.02 (6.1)	L = 102.75 (33.9) H = 115.15 (36.8)	L = 91.6 (13.40) H = 99 (13.30)	L = 136.5 (10.4) H = 133.4 (10.2)	Mean (SD)
Mirmiran 2015 (Iran) [103]	1683 (927/756)	L = 36.3 (13.3) H = 41.3 (13.8)	General population	Trichopoulou MDS—8 points [21]	L = 1 H = 0.74 [0.48–1.13]	L = 1 H = 0.82 [0.48–1.40] *	L = 1 H = 0.81 [0.56–1.17] *	L = 1 H = 1.01 [0.73–1.39]	L = 1 H = 0.86 [0.64–1.22]	OR
Mziwira 2015 (Morocco) [104]	90 (90/0)	39.9 (0.66)	General non-pregnant population	Specific MDS- 0%–100% [42]	L = 1 H = 0.54 [0.13–2.27]	L = 1 H = 0.29 [0.02–3.02]	L = 1 H = 0.47 [0.04–4.94]	L = 1 H = 0.27 [0.05–1.49]	L = 1 H = 0.77 [0.19–3.15]	OR
Roldan 2019 (Spain) [105]	107 (58/49)	61.16 (23)	Overweight/Obese T2DM patients with poor glycemic control	PREDIMED MEDAS score—14 points [33]	Νo info	L = 48.29 H = 52.45 *	L = 223.56 H = 171.23 **	L = 201.14 H = 132.88 *	Νo info	Mean
Rumawas 2009 (US) [106]	1069 (608/461)	L = 52.4 (9.9) H = 54.8 (9.6)	Non-diabetic general population	The MSDPS—100 points [36]	L = 98.5 H = 97.1 **	L = 53.3 H = 54 *	L = 114 H = 103 **	L= 98.5 H = 97.1 *	L = 122 H = 121	Geometric mean
Steffen 2014 (US) [44]	865 (511/354)	L = 24.3 H = 25.7	General population	Modified Trichopoulou MDS—22 points [21]	L = 59.4% H = 41.9% **	L = 68.4% H = 59.3% *	L = 37.3% H = 21.6% **	L= 21.3% H = 19.1% *	L= 49.2% H = 40.4% *	%Prevalence
Tortosa 2007 (Spain) [107]	1040 (No info)	No info	Graduate students	Trichopoulou MDS—9 points [21]	L = 82.5 (12) H = 82 (12) *	L = 63.8 (15) H = 64.1 (19) *	L = 80.0 (38) H = 78 (40)	L = 86.1 (11) H = 87.3 (17)	L = 112.5 (14) H = 113.3 (13)	Mean (SD)
Yang 2014 (US) [43]	395 (0/395)	L = 38.2 (8.6) H = 37.1 (8.4)	General population	Study Specific MDS—42 points [43]	No info	L = 41.7 (1.3) H = 46.6 (1.3)	L = 140.4 (1.8) H = 115.8 (1.8)	L = 93.2 (1.2) H = 91.1 (1.2)	L = 122.4 (12.6) H = 122.8 (13.3)	Geometric mean (SD)

* *p* < 0.05, ** *p* < 0.001. ^1^: Age range. Variables are displayed as mean (SD), OR [95% Confidence Interval]. CKD: Chronic Kidney Disease, F: Female, FBG: Fasting Blood Glucose, FFQ: Food Frequency Questionnaire, H: High Adherence, HDL: High-Density Lipoprotein, L: Low Adherence, M: Male, M: Moderate Adherence, MD: Mediterranean Diet, MEDAS: Mediterranean Diet Adherence Screener, MDS: Mediterranean Diet Score, MSDPS: Mediterranean-Style Dietary Pattern Score, OR: Odds Ratio, SBP: Systolic Blood Pressure, SD: Standard Deviation, T2DM: Type 2 Diabetes Mellitus, TG: Triglycerides and WC: Waist circumference.

**Table 3:** Characteristics of studies included in the meta-analysis.

Study ID (Country)	No Participants (F/M)	Age (Years)	Population	MD Assessment Tool
Abiemo 2013 (US) [45]	2440 (1305/1135)	L = 60.0 (10.3) H = 63.0 (10.3)	General population	Study Specific Alternate MDS—10 points [45]
Ahmad 2018 (US) [58]	16,623 (16,623/0)	L = 52.6 (6.7) H = 54.9 (8.1)	General population	Trichopoulou MDS—9 points [21]
Ahmed 2020 (US) [59]	224 (133/91)	L = 56.2 (12.6) H = 66.7 (11.6)	Community-dweling adults	Sofi MDS—12 points [32]
Asghari 2016 (Iran) [60]	622 (308/314)	L = 43.0 (9.1) H = 43.7 (9.7)	Subjects without CKD	Trichopoulou MDS—8 points [108]
Baratta 2017 (Italy) [61]	148 (47/101)	L = 51.7 (11.3) H = 57.7 (11.9)	Outpatients presenting with T2DM, HBP, Overweight/Obese, Dyslipedemia or MetS	Short MDS—9 points [34]
Bondia-Pons 2009 (Spain) [62]	70 (41/29)	47 (15.3)	General population	MD Quality Index—14 point % adherence [37]
Campanella 2020 (Italy) [63]	2387 (1183/1204)	L = 45.5(15.5) H = 54.6 (15.5)	General population	Relative MD system—18 points [38]
Dai 2008 (US) [64]	194 (0/194)	L = 53.8 (0.3) H = 54.8 (0.3)	Middle aged twins who have served in the Vietman War	Trichopoulou MDS—9 points [21]
Esposito 2009 (Italy) [65]	475 (232/243)	L = 58.0 (7.0) H = 58.3 (7.0)	T2DM patients	Trichopoulou MDS—9 points [21]
Gardener 2015 (US) [66]	543 (308/235)	L = 69.0 (8.0) H = 65.0 (9.0)	Population never diagnosed with stroke	Trichopoulou MDS—9 points [21]
Giraldi 2020 (Italy) [67]	209 (61/148)	L = 41.7 (13.3) H = 49.9 (16.4)	Patients with NAFLD	Sofi MDS—12 points [32]
Giugliano 2010a (Italy) [69]	315 (315/0)	L = 57.7 (6.7) H = 58.0 (6.8)	T2DM patients	Trichopoulou MDS—9 points [21]
Giugliano 2010b (Italy) [68]	288 (0/288)	L = 54.7 (6.9) H = 58.7 (7.0)	T2DM patients	Trichopoulou MDS- 9 points [21]
Granado-Casas 2020 (Spain) [70]	92 (52/40)	L = 41.9 (10.6) H = 45.1 (10.9)	T1DM patients	Trichopoulou MDS—9 points [21]
Grosso 2015 (Poland) [46]	4678 (2408/2270)	45–69 *	General population	Modified Panagiotakos MDS—60 points [31]
Hu 2013 (Spain) [71]	7305 (4188/3117)	L = 67.2 (6.2) H = 67.0 (6.2)	Adults with high risk of CVD, with T2DM or at least 3/6 CVD risk factors	PREDIMED MEDAS Score—14 points [33]
Izadi 2016 (Iran) [72]	325 (325/0)	L = 28.0 (6.2) H = 27.2 (5.2)	Pregnant carrying singleton fetuses with/without GDM	Trichopoulou MDS—9 points [21]
Jalilpiran 2020 (Iran) [73]	357 (0/357)	L = 66.5 (6.7) H = 63.3 (5.8)	General population	Trichopoulou MDS—9 points [21]
Jayedi 2019 (Iran) [74]	131 (131/0)	L = 54.7 (6.8) H = 54.9 (7.5)	Females with prevalent T2DM or with history of 3–10 yrs T2DM and with/without DN	Trichopoulou MDS—9 points [21]
Köroğlu 2020 (Turkey) [75]	25 (0/25)	18–65 *	Patients with lower limb amputation	PREDIMED MEDAS Score—14 points [33]
Kwon 2020 (Korea) [76]	148 (84/64)	L = 43.6 (9.1) H = 53.3 (8.3)	General Population	PREDIMED MEDAS Score—14 points [33]
Lavados 2020 (Leu) [77]	368 (158/210)	L = 67.2 (18.7) H = 69.9 (16.9)	Patients with acute ischemic stroke	PREDIMED MEDAS Score—14 points [33]
Leu 2019 (Taiwan) [78]	1400 (807/593)	L = 48.4 (12.7) H = 50.6 (11.4)	General Population	Trichopoulou MDS—9 points [21]
Mateo-Gallego 2017 (Spain) [79]	1016 (54/962)	L = 50.9 (4.0) H = 51.7 (3.7)	Employees of car assembly plant	Trichopoulou MDS—9 points [21]
Molina-Leyva 2018 (Spain) [80]	25 (No info)	L = 43.7 (10.9) H = 50.8 (13.5)	Patients with psoriasis	PREDIMED MEDAS Score—14 points [33]
Moradi 2020 (Iran) [81]	153 (95/58)	L = 64.7 (9.3) H = 67.2 (9.8)	Diabetic patients with nephropathy	Trichopoulou MDS—9 points [21]
Mosconi 2014 (US) [82]	52 (37/15)	L = 53.0 (13) H = 55.0 (12)	Cognitive-normal individuals	Study Specific MDS—9 points [82]
Park 2016 (US) [83]	1034 (572/462)	L = 40.8 (0.9) H = 40.8 (1.3)	Metabolicaly healthy and unhealthy obese population	Panagiotakos MDS—55 points [31]
Peñalvo 2015 (Spain) [84]	516 (18/498)	L = 50.8 (3.8) H = 51.5 (3.4)	General population	MEDAS Score [33] Alternative MD index [41]
Pocovi-Gerardino 2020 (Spain) [85]	159 (143/16)	L = 38.6 (9.7) H = 28.3 (12.8)	Patients with SLE	PREDIMED MEDAS Score—14 points [33]
Ruiz-Cabello 2016 (Spain) [86]	118 (118/0)	L = 52.0 (4.8) H = 52.9 (4.1)	Peri- and menopausal females	Panagiotakos MDS—55 points [31]
Salas-Huetos 2019 (Spain) [87]	57 (0/57)	L = 24.1 (4.5) H = 26.3 (4.8)	Healthy subjects	Trichopoulou MDS—9 points [21]
Sotos-Prieto 2014 (UK) [88]	10,359 (5593/4766)	L = 59.0 (9.4) H = 59.3 (9.3)	General population	Trichopoulou MDS—9 points [21]
Tuttolomondo 2015 (Italy) [89]	288 (162/126)	L = 72.9 (14.8) H = 72.4 (13.2)	Patients with ischemic heart disease	Trichopoulou MDS—9 points [21]
Tuttolomondo 2020 (Italy) [90]	409 (250/159)	L = 70.2 (12.6) H = 72.0 (10.4)	Patients with congestive heart failure	Trichopoulou MDS—9 points [21]
Tzima 2007 (Greece) [91]	1040 (333/707)	L = 55.0 (13) H = 35.0 (10)	Obese and Overweight population	Panagiotakos MDS—55 points [31]
Veglia 2019 (Finland, Sweden, Netherlands, France, Italy) [47]	1835 (980/855)	L = 64.8 (5.4) H = 63.9 (5.7)	Patients with >3 vascular risk factors	Study Specific MDS—7 points [47]
Veissi 2016 (Iran) [92]	157 (104/53)	L = 54.3 (9.9) H = 54.6 (8.9)	T2DM patients	Study Specific MDS—4 points [92]
Viscogliosi 2013 (Italy) [93]	55 (33/22)	L = 59.6 (10.2) H = 60.0 (9.4)	High CVD risk population	PREDIMED MEDAS Score—14 points [33]
Vitale 2018 (Italy) [49]	1539 (606/933)	No info	T2DM patients with HbA1c 7%–9%	Modified Trichopoulou MDS—18 points [21]
Zupo 2020 (Italy) [94]	324 (228/96)	L = 38.0 (13.1) H = 42.5 (13.1)	General population	PREDIMED MEDAS Score—14 points [33]

* Age range. Variables are displayed as mean (SD). CKD: Chronic Kidney Disease, CVD: Cardiovascular Diseases, DN: Diabetic Nephropathy, F: Female, GDM: Gestational Diabetes Mellitus, H: High Adherence, HBP: High Blood Pressure, L: Low Adherence: M: Male, MDS: Mediterranean Diet Score, MEDAS: Mediterranean Diet Adherence Screener, MetS: Metabolic Syndrome, NAFLD: Non-Alcoholic Fatty Liver Disease, SLE: Systemic Lupus Erythematosus, T1DM: Type 1 Diabetes Mellitus and T2DM: Type 2 Diabetes Mellitus.

## Data Availability

Not Applicable.

## Supplementary Materials

The following are available online at https://www.mdpi.com/article/10.3390/nu13051514/s1, Supplementary File S1: MOOSE checklist, Supplementary File S2: Search Strategy, Supplementary File S3: Quality of Studies according to the New Castle Ottawa Scale, Supplementary Figure S1a–e: Funnel plots of studies included in our meta-analysis regarding each parameter of MetS, Supplementary Figure S2: Subgroup analysis based on the quality of studies regarding WC, Supplementary Figure S3: Subgroup analysis based on the quality of studies regarding HDL cholesterol, Supplementary Figure S4: Subgroup analysis based on the quality of studies regarding serum TG, Supplementary Figure S5: Subgroup analysis based on the quality of studies regarding FBG Supplementary Figure S6: Subgroup analysis based on the quality of studies regarding SBP.

## Abbreviations

**BP**	Blood Pressure
**CI**	Confidence Interval
**CVD**	Cardiovascular Disease
**DBP**	Diastolic Blood Pressure
**FFQs**	Food Frequency Questionnaires
**FG**	Fasting Glucose
**GLP-1**	Glucagon-Like Peptide-1
**HbA1c**	Glycohemoglobin
**HDL**	High-Density Lipoprotein
**IDF**	International Diabetes Federation
**MD**	Mediterranean Diet
**MDS**	Mediterranean Diet Score
**MEDAS**	Mediterranean Diet Adherence Screener
**MetS**	Metabolic Syndrome
**MSDPS**	Mediterranean-Style Dietary Pattern Score
**MOOSE**	Meta-analyses Of Observational Studies in Epidemiology
**N-3 FAs**	Omga-3-Fatty Acids
**NAFLD**	Non-Alcoholic Fatty Liver Disease
**NCDs**	Non-Communicable Diseases
**NCEP ATP III**	National Cholesterol Program in Adult Treatment Panel III
**NHNES**	National Health and Nutrition Examination Survey
**NOS**	New Castle Ottawa Scale
**OR**	Odds Ration
**RCT**	Randomized Controlled Trial
**SD**	Standard Deviation
**SBP**	Systolic Blood Pressure
**SMD**	Standardized Mean Difference
**T2DM**	Type 2 Diabetes Mellitus
**TG**	Triglycerides
**UK**	United Kingdom
**US**	United States
**WC**	Waist Circumference
**WHO**	World Health Organization
