# Supplementary material for: Impact of the Level of Adherence to Mediterranean Diet on the Parameters of Metabolic Syndrome: A Systematic Review and Meta-Analysis of Observational Studies
Source: Nutrients. 2021 Apr 30;13(5):1514. doi: 10.3390/nu13051514 (PMC8146502; doi:10.3390/nu13051514)
Supplement: Supplementary file 1 [file nutrients-13-01514-s001.zip › Supplementary Figure S1a-e.pdf]

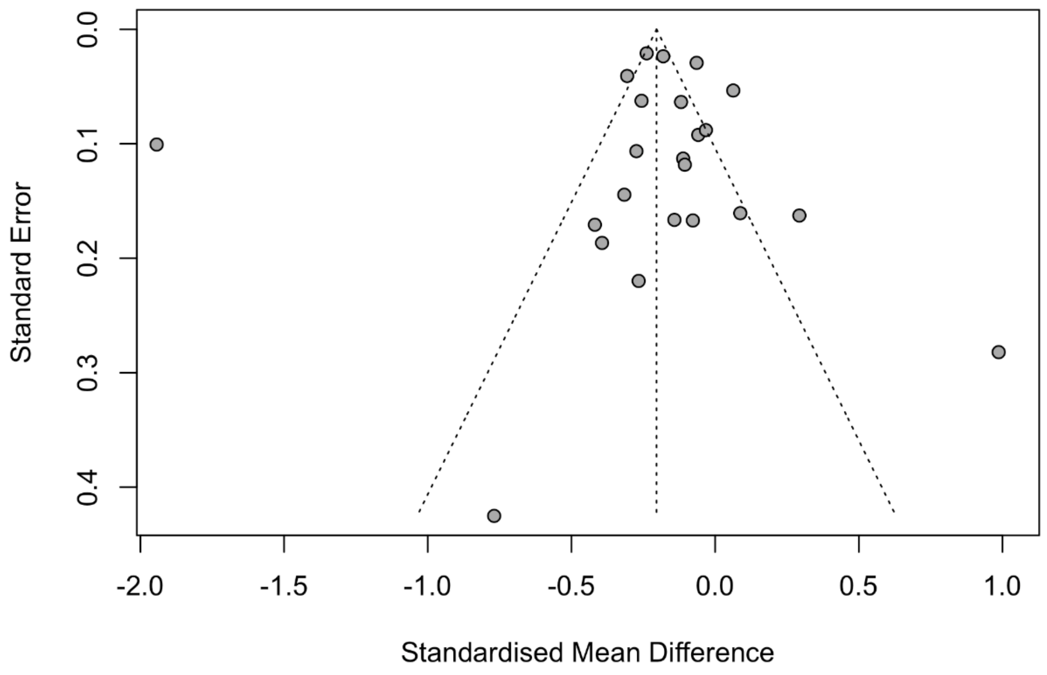

Supplementary Figure S1a: Funnel plot of studies referred to WC

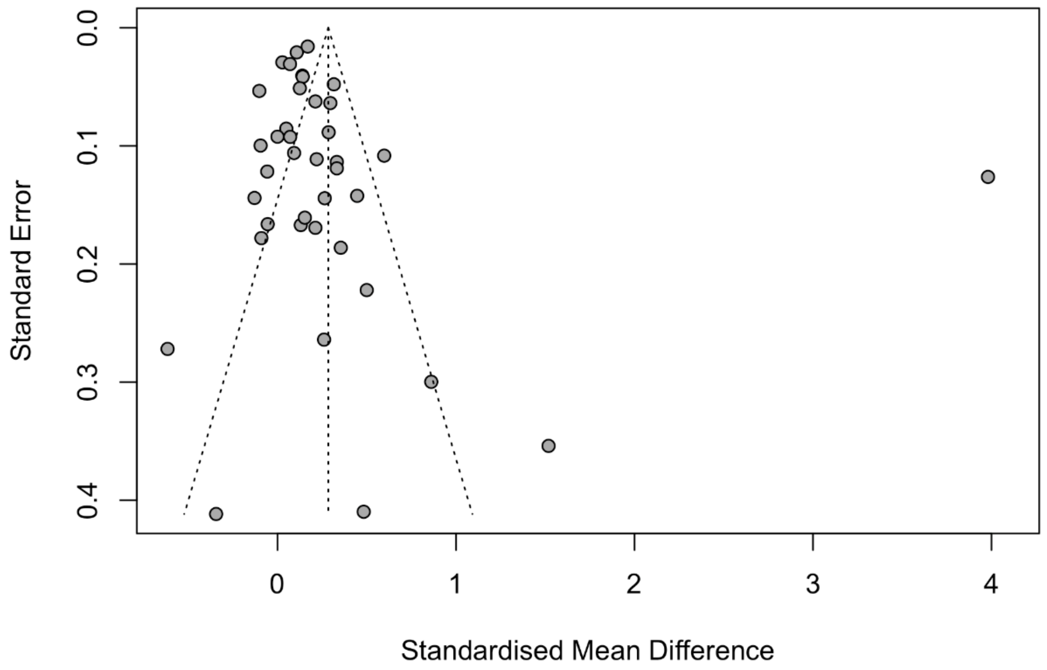

Supplementary Figure S1b: Funnel plot of studies referred to HDL cholesterol

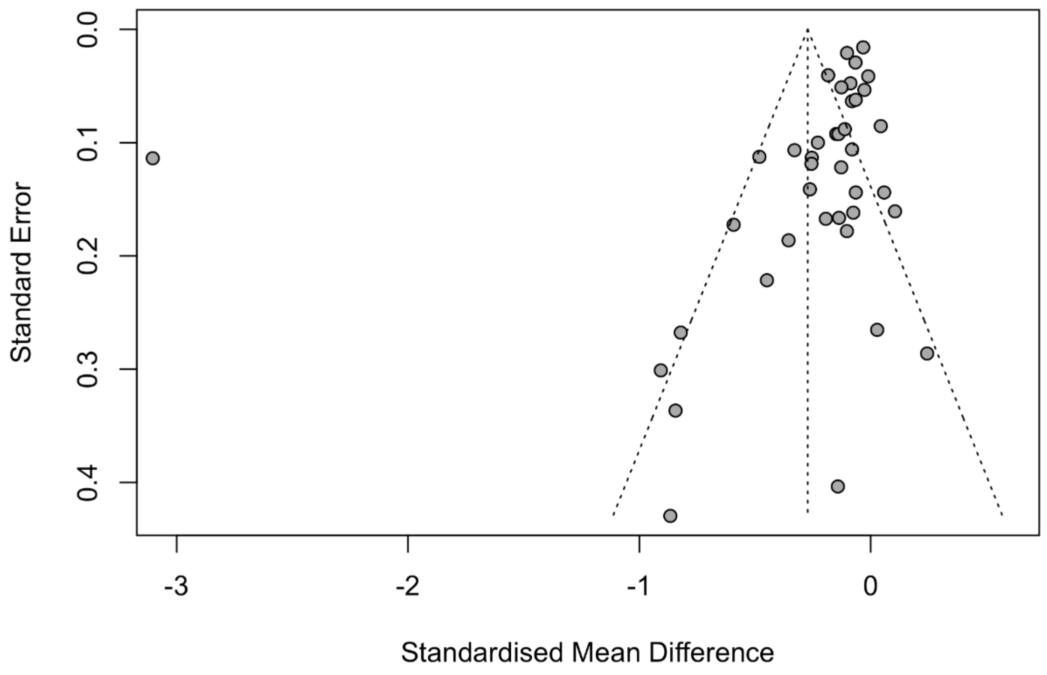

Supplementary Figure S1c: Funnel plot of studies referred to serum Triglycerides

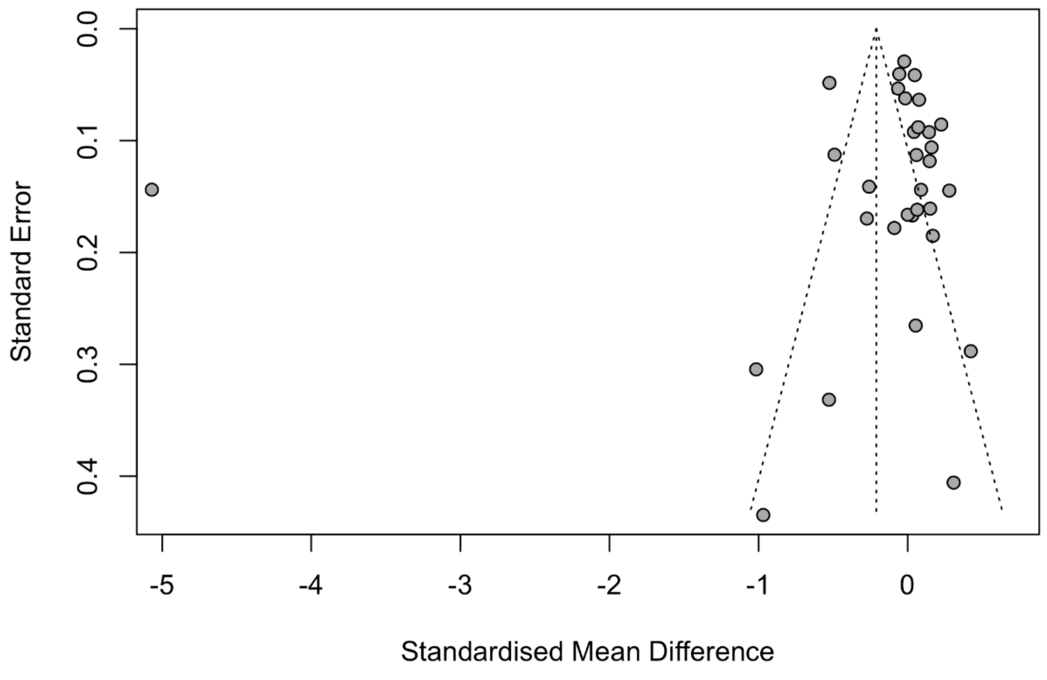

Supplementary Figure S1d: Funnel plot of studies referred to serum Fasting Blood Glucose

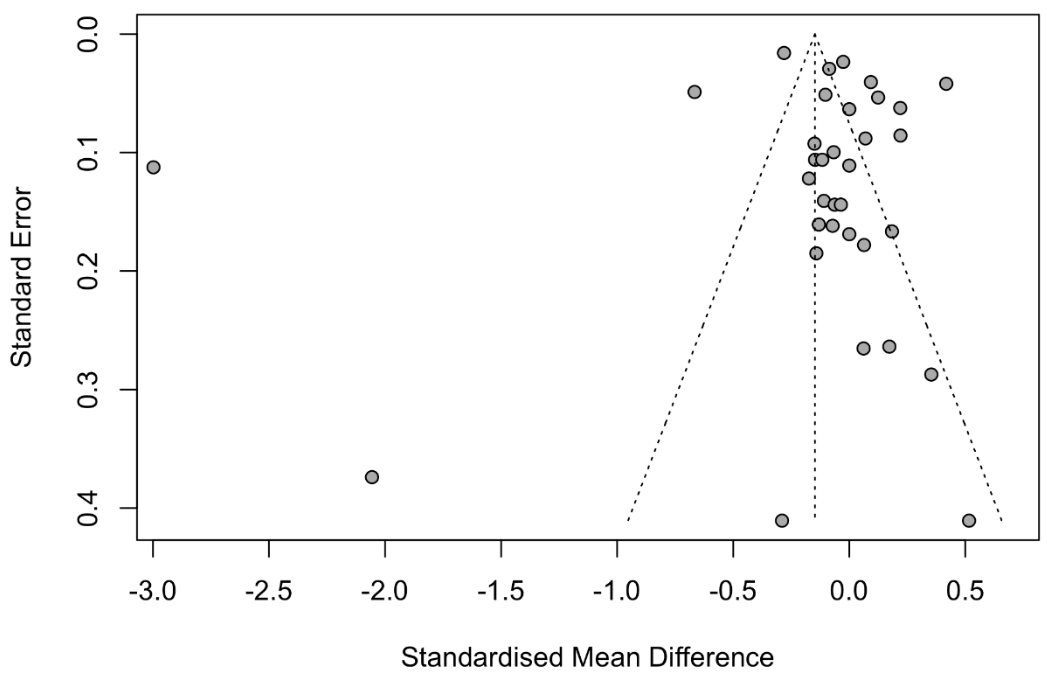

Supplementary Figure S1e: Funnel plot of studies referred to serum Systolic Blood Pressure
