# Supplementary material for: Impact of the Level of Adherence to Mediterranean Diet on the Parameters of Metabolic Syndrome: A Systematic Review and Meta-Analysis of Observational Studies
Source: Nutrients. 2021 Apr 30;13(5):1514. doi: 10.3390/nu13051514 (PMC8146502; doi:10.3390/nu13051514)
Supplement: Supplementary file 1 [file nutrients-13-01514-s001.zip › Supplementary Figure S2_WC subgroup.pdf]

| Study | High Adherence Total | High Adherence Mean | High Adherence SD | Low Adherence Total | Low Adherence Mean | Low Adherence SD | Hedges' g | 95% CI | Weight |
|---|---|---|---|---|---|---|---|---|---|
| NOS = ≥7 | | | | | | | | | |
| Abiemo 2013 | 1200 | 94.90 | 13.2000 | 1240 | 99.20 | 14.8000 | -0.31 | [-0.39; -0.23] | 4.8% |
| Esposito 2009 | 214 | 98.10 | 12.0000 | 261 | 98.80 | 11.8000 | -0.06 | [-0.24; 0.12] | 4.6% |
| Giugliano 2010 (F) | 149 | 96.00 | 10.8000 | 166 | 97.20 | 10.7000 | -0.11 | [-0.33; 0.11] | 4.5% |
| Giugliano 2010 (M) | 133 | 99.30 | 10.9000 | 155 | 100.50 | 11.7000 | -0.11 | [-0.34; 0.13] | 4.5% |
| Granado-Casas 2020 | 32 | 85.80 | 16.0000 | 60 | 89.70 | 13.7000 | -0.27 | [-0.70; 0.16] | 3.9% |
| Grosso 2015 | 2218 | 92.10 | 12.3000 | 2460 | 92.90 | 12.5000 | -0.06 | [-0.12; -0.01] | 4.8% |
| Jalilpiran 2020 | 171 | 95.00 | 7.8000 | 186 | 97.20 | 8.2000 | -0.27 | [-0.48; -0.07] | 4.5% |
| Kwon 2020 | 63 | 78.60 | 10.0000 | 85 | 80.10 | 10.9000 | -0.14 | [-0.47; 0.18] | 4.2% |
| Mateo-Gallego 2017 | 582 | 96.40 | 9.8200 | 434 | 97.60 | 10.5000 | -0.12 | [-0.24; 0.01] | 4.7% |
| Moradi 2020 | 74 | 103.50 | 10.4000 | 79 | 100.20 | 11.9000 | 0.29 | [-0.03; 0.61] | 4.2% |
| Park 2016 | 513 | 107.30 | 15.3000 | 521 | 110.90 | 12.7000 | -0.26 | [-0.38; -0.13] | 4.7% |
| Peñalvo 2015 | 258 | 97.70 | 8.9000 | 258 | 98.00 | 9.7000 | -0.03 | [-0.20; 0.14] | 4.6% |
| Salas-Huetos 2019 | 27 | 84.60 | 6.8000 | 30 | 78.40 | 5.6000 | 0.99 | [ 0.43; 1.54] | 3.5% |
| Sotos-Prieto 2014 | 3435 | 86.40 | 12.3000 | 6924 | 89.30 | 12.1000 | -0.24 | [-0.28; -0.20] | 4.8% |
| Tzima 2007 | 139 | 77.70 | 10.6000 | 901 | 101.90 | 12.7000 | -1.94 | [-2.14; -1.75] | 4.6% |
| Random effects model | 9208 | | | 13760 | | | -0.19 | [-0.48; 0.10] | 66.8% |
| Heterogeneity: $I^2$ = 96%, $\tau^2$ = 0.3049, $p$ < 0.01 | | | | | | | | | |
| NOS = <7 | | | | | | | | | |
| Baratta 2017 | 91 | 103.00 | 11.4000 | 57 | 108.10 | 13.2000 | -0.42 | [-0.75; -0.08] | 4.2% |
| Giraldi 2020 | 46 | 100.70 | 10.0000 | 163 | 101.60 | 12.0000 | -0.08 | [-0.40; 0.25] | 4.2% |
| Hu 2013 | 3956 | 99.60 | 10.7000 | 3349 | 101.50 | 10.3000 | -0.18 | [-0.23; -0.13] | 4.8% |
| Köroğlu 2020 | 10 | 93.50 | 5.2000 | 15 | 104.00 | 16.4000 | -0.77 | [-1.60; 0.06] | 2.6% |
| Leu 2019 | 703 | 80.60 | 9.0000 | 697 | 80.00 | 10.0000 | 0.06 | [-0.04; 0.17] | 4.7% |
| Ruiz-Cabello 2016 | 54 | 91.30 | 11.0000 | 64 | 95.70 | 11.2000 | -0.39 | [-0.76; -0.03] | 4.1% |
| Veissi 2016 | 87 | 98.90 | 10.4000 | 70 | 98.00 | 10.0000 | 0.09 | [-0.23; 0.40] | 4.3% |
| Zupo 2020 | 265 | 107.50 | 12.6000 | 59 | 111.70 | 15.9000 | -0.32 | [-0.60; -0.03] | 4.3% |
| Random effects model | 5212 | | | 4474 | | | -0.17 | [-0.32; -0.02] | 33.2% |
| Heterogeneity: $I^2$ = 75%, $\tau^2$ = 0.0259, $p$ < 0.01 | | | | | | | | | |
| **Random effects model** | **14420** | | | **18234** | | | **-0.20** | **[-0.40; -0.01]** | **100.0%** |

Heterogeneity: $I^2$ = 95%, $\tau^2$ = 0.2058, $p$ < 0.01

Residual heterogeneity: $I^2$ = 95%, $p$ < 0.01

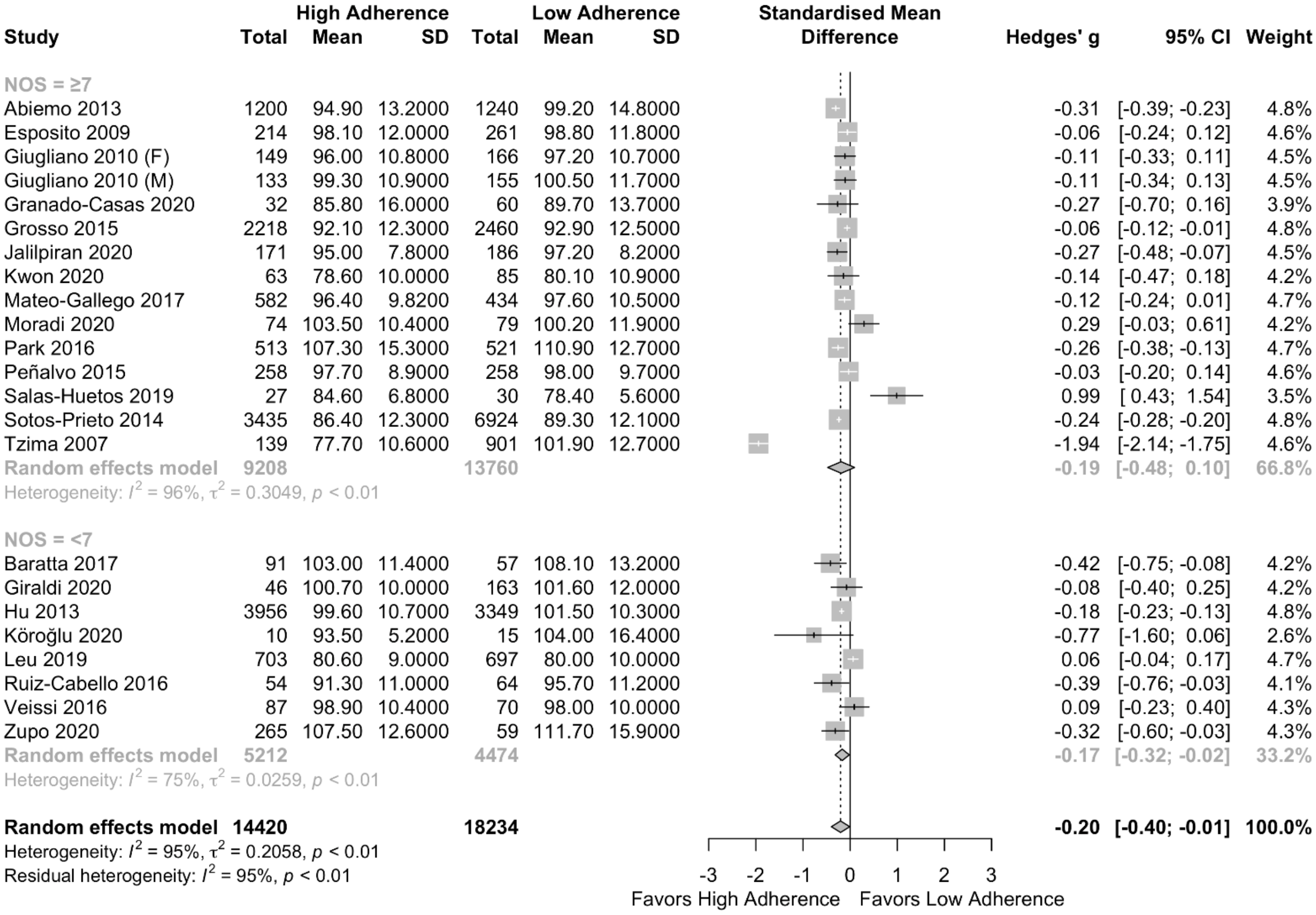


**Supplementary Figure S2:** Subgroup analysis based on the quality of studies regarding Waist Circumference (cm)
