# Supplementary material for: Impact of the Level of Adherence to Mediterranean Diet on the Parameters of Metabolic Syndrome: A Systematic Review and Meta-Analysis of Observational Studies
Source: Nutrients. 2021 Apr 30;13(5):1514. doi: 10.3390/nu13051514 (PMC8146502; doi:10.3390/nu13051514)
Supplement: Supplementary file 1 [file nutrients-13-01514-s001.zip › Supplementary Figure S3_HDL subgroup.pdf]

Study	High Adherence Total	High Adherence Mean	High Adherence SD	Low Adherence Total	Low Adherence Mean	Low Adherence SD	Hedges' g	95% CI	Weight
**NOS = ≥7**									
Abiemo 2013	1200	53.00	15.3000	1240	50.90	14.8000	0.14	[0.06; 0.22]	2.7%
Ahmad 2018	6483	54.20	14.6000	10140	51.80	13.9000	0.17	[0.14; 0.20]	2.7%
Ahmed 2020	77	58.60	15.6000	147	51.70	15.3000	0.45	[0.17; 0.73]	2.6%
Asghari 2016	204	42.10	10.0000	418	41.60	10.2000	0.05	[-0.12; 0.22]	2.7%
Bondia-Pons 2009	59	54.10	5.4000	11	45.20	7.7000	1.52	[0.82; 2.21]	2.1%
Dai 2008	90	37.70	9.5000	104	38.90	9.1000	-0.13	[-0.41; 0.15]	2.6%
Esposito 2009	214	42.50	8.9000	261	42.50	8.9000	0.00	[-0.18; 0.18]	2.7%
Gardener 2015	372	46.00	14.0000	171	45.00	14.0000	0.07	[-0.11; 0.25]	2.7%
Giugliano 2010 (F)	149	47.00	9.0000	166	44.00	9.0000	0.33	[0.11; 0.56]	2.6%
Giugliano 2010 (M)	133	47.00	9.0000	155	44.00	9.0000	0.33	[0.10; 0.57]	2.6%
Granado-Casas 2020	32	67.50	10.7000	60	59.50	18.0000	0.50	[0.06; 0.94]	2.4%
Grosso 2015	2218	55.70	14.3000	2460	55.30	14.7000	0.03	[-0.03; 0.08]	2.7%
Izadi 2016	165	47.70	8.9000	160	45.70	9.3000	0.22	[0.00; 0.44]	2.6%
Jalilpiran 2020	171	51.90	7.8000	186	47.10	8.2000	0.60	[0.39; 0.81]	2.6%
Jayedi 2019	78	44.70	8.8000	53	45.50	8.9000	-0.09	[-0.44; 0.26]	2.5%
Kwon 2020	63	59.20	14.0000	85	60.00	14.9000	-0.05	[-0.38; 0.27]	2.6%
Lavados 2020	151	48.60	14.8000	217	47.20	15.2000	0.09	[-0.11; 0.30]	2.6%
Mateo-Gallego 2017	582	55.40	11.9000	434	51.90	11.7000	0.30	[0.17; 0.42]	2.7%
Park 2016	513	47.10	23.1000	521	43.10	13.3000	0.21	[0.09; 0.33]	2.7%
Peñalvo 2015	258	54.80	11.5000	258	51.50	11.5000	0.29	[0.11; 0.46]	2.7%
Pocovi-Gerardino 2020	143	59.10	17.1000	16	54.60	17.7000	0.26	[-0.26; 0.78]	2.3%
Salas-Huetos 2019	27	54.20	8.6000	30	59.70	9.0000	-0.62	[-1.15; -0.08]	2.3%
Sotos-Prieto 2014	3435	53.40	11.6000	6924	51.80	16.2000	0.11	[0.07; 0.15]	2.7%
Tzima 2007	139	47.60	0.4000	901	45.30	0.6000	3.98	[3.73; 4.23]	2.6%
Veglia 2019	737	51.00	0.4000	1098	47.60	13.9000	0.32	[0.22; 0.41]	2.7%
**Random effects model**	**17693**			**26216**			**0.36**	**[0.03; 0.68]**	**64.9%**
Heterogeneity: I^2 = 98%, τ^2 = 0.6685, p < 0.01									
**NOS = <7**									
Baratta 2017	91	49.10	12.3000	57	46.60	10.7000	0.21	[-0.12; 0.54]	2.6%
Campanella 2020	1001	52.60	13.9000	1386	50.70	13.1000	0.14	[0.06; 0.22]	2.7%
Giraldi 2020	46	48.60	14.5000	163	46.50	16.4000	0.13	[-0.20; 0.46]	2.6%
Hu 2013	2471	53.40	12.8000	1853	52.50	12.8000	0.07	[0.01; 0.13]	2.7%
Köroğlu 2020	10	38.00	6.1000	15	40.60	8.0000	-0.34	[-1.15; 0.46]	2.0%
Leu 2019	703	41.90	12.3000	697	43.20	13.2000	-0.10	[-0.21; 0.00]	2.7%
Molina-Leyva 2018	14	58.90	9.7000	11	51.40	19.9000	0.48	[-0.32; 1.29]	2.0%
Ruiz-Cabello 2016	54	64.50	17.6000	64	57.90	19.2000	0.35	[-0.01; 0.72]	2.5%
Tuttolomondo 2015	180	47.50	18.3000	108	48.50	15.9000	-0.06	[-0.30; 0.18]	2.6%
Tuttolomondo 2020	179	45.50	17.7000	230	47.10	16.2000	-0.09	[-0.29; 0.10]	2.7%
Veissi 2016	87	44.80	10.0000	70	43.30	9.4000	0.15	[-0.16; 0.47]	2.6%
Viscogliosi 2013	18	60.00	14.6000	37	48.00	13.3000	0.86	[0.27; 1.45]	2.3%
Vitale 2018	705	46.80	12.4000	834	45.30	11.6000	0.13	[0.02; 0.23]	2.7%
Zupo 2020	265	52.10	13.4000	59	48.50	14.3000	0.26	[-0.02; 0.55]	2.6%
**Random effects model**	**5824**			**5584**			**0.09**	**[0.01; 0.17]**	**35.1%**
Heterogeneity: I^2 = 60%, τ^2 = 0.0104, p < 0.01									
**Random effects model**	**23517**			**31800**			**0.28**	**[0.07; 0.50]**	**100.0%**
Heterogeneity: I^2 = 96%, τ^2 = 0.4499, p < 0.01									
Residual heterogeneity: I^2 = 96%, p < 0.01									

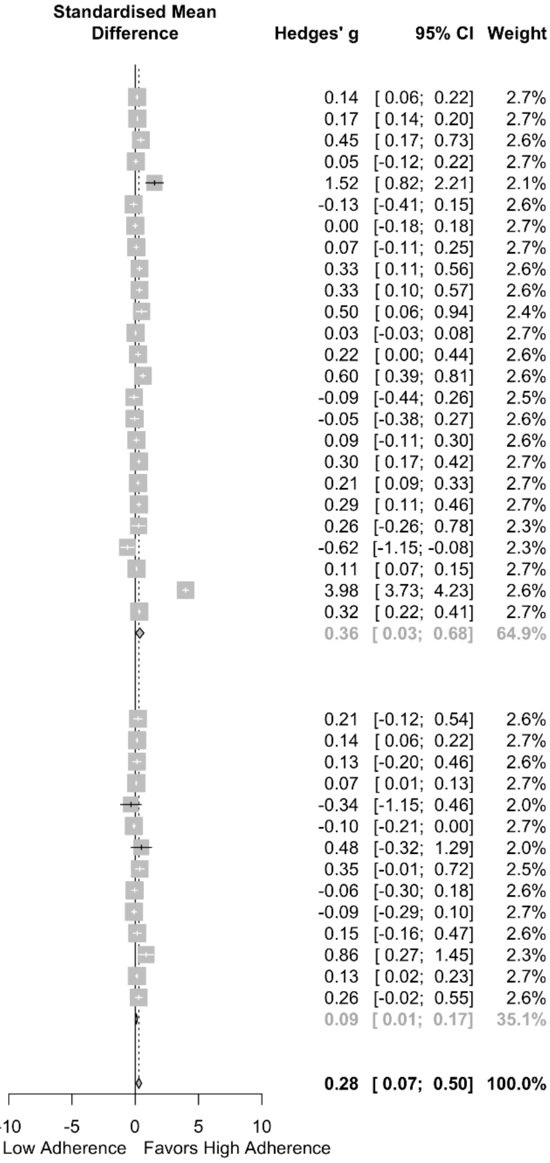

Supplementary Figure S3: Subgroup analysis based on the quality of studies regarding High Density Lipoprotein Cholesterol (mg/dL)
