# Supplementary material for: Impact of the Level of Adherence to Mediterranean Diet on the Parameters of Metabolic Syndrome: A Systematic Review and Meta-Analysis of Observational Studies
Source: Nutrients. 2021 Apr 30;13(5):1514. doi: 10.3390/nu13051514 (PMC8146502; doi:10.3390/nu13051514)
Supplement: Supplementary file 1 [file nutrients-13-01514-s001.zip › Supplementary Figure S4_TG Supgroup.pdf]

Study	High Adherence Total	High Adherence Mean	High Adherence SD	Low Adherence Total	Low Adherence Mean	Low Adherence SD	Hedges' g	95% CI	Weight
NOS = ≥7									
Abiemo 2013	1200	118.00	66.0000	1240	131.00	75.0000	-0.18	[-0.26; -0.10]	2.7%
Ahmad 2018	6483	123.30	63.8000	10140	125.40	65.9000	-0.03	[-0.06; 0.00]	2.7%
Ahmed 2020	77	117.80	60.2000	147	135.50	70.5000	-0.26	[-0.54; 0.01]	2.6%
Asghari 2016	204	158.00	91.7000	418	153.80	97.5000	0.04	[-0.12; 0.21]	2.7%
Bondia-Pons 2009	59	91.20	17.7000	11	106.30	17.7000	-0.84	[-1.50; -0.18]	1.9%
Dai 2008	90	182.50	99.6000	104	176.60	102.0000	0.06	[-0.22; 0.34]	2.5%
Esposito 2009	214	159.40	61.1000	261	168.30	58.5000	-0.15	[-0.33; 0.03]	2.7%
Gardener 2015	372	135.00	79.0000	171	146.00	79.0000	-0.14	[-0.32; 0.04]	2.7%
Giugliano 2010 (F)	149	156.00	60.0000	166	172.00	65.0000	-0.25	[-0.48; -0.03]	2.6%
Giugliano 2010 (M)	133	153.00	59.0000	155	168.00	58.0000	-0.26	[-0.49; -0.02]	2.6%
Grosso 2015	2218	137.30	64.7000	2460	141.70	69.0000	-0.07	[-0.12; -0.01]	2.7%
Granado-Casas 2020	32	57.90	16.6000	60	67.50	23.3000	-0.45	[-0.88; -0.01]	2.3%
Izadi 2016	165	131.70	55.9000	160	161.50	67.4000	-0.48	[-0.70; -0.26]	2.6%
Jalilpiran 2020	171	123.70	39.2000	186	136.70	39.6000	-0.33	[-0.54; -0.12]	2.6%
Jayedi 2019	78	163.10	70.7000	53	169.90	58.0000	-0.10	[-0.45; 0.25]	2.5%
Kwon 2020	63	101.90	60.4000	85	110.10	58.9000	-0.14	[-0.46; 0.19]	2.5%
Lavados 2020	151	130.70	111.9000	217	138.40	82.6000	-0.08	[-0.29; 0.13]	2.6%
Mateo-Gallego 2017	582	146.00	104.0000	434	154.00	92.3000	-0.08	[-0.20; 0.04]	2.7%
Moradi 2020	74	254.30	59.2000	79	258.70	57.5000	-0.08	[-0.39; 0.24]	2.5%
Mosconi 2014	20	96.00	49.0000	32	86.00	34.0000	0.24	[-0.32; 0.80]	2.1%
Park 2016	513	164.80	176.6000	521	174.30	109.0000	-0.06	[-0.19; 0.06]	2.7%
Peñalvo 2015	258	145.00	98.3000	258	156.00	98.3000	-0.11	[-0.28; 0.06]	2.7%
Pocovi-Gerardino 2020	143	91.60	43.9000	16	128.30	49.8000	-0.82	[-1.35; -0.30]	2.2%
Salas-Huetos 2019	27	71.40	22.7000	30	70.80	19.5000	0.03	[-0.49; 0.55]	2.2%
Sotos-Prieto 2014	3435	134.60	53.1000	6924	139.00	37.2000	-0.10	[-0.14; -0.06]	2.7%
Tzima 2007	139	129.00	11.0000	901	146.00	4.0000	-3.10	[-3.33; -2.88]	2.6%
Veglia 2019	737	117.60	58.7000	1098	123.00	63.8000	-0.09	[-0.18; 0.01]	2.7%
Random effects model	17787			26327			-0.29	[-0.52; -0.05]	68.8%
Heterogeneity: $I^2 = 97\%$, $\tau^2 = 0.3579$, $p < 0.01$									
NOS = <7									
Baratta 2017	91	127.70	42.2000	57	167.20	92.8000	-0.59	[-0.93; -0.25]	2.5%
Campanella 2020	1001	120.50	80.6000	1386	121.40	91.2000	-0.01	[-0.09; 0.07]	2.7%
Giraldi 2020	46	142.00	79.0000	163	158.10	83.8000	-0.19	[-0.52; 0.13]	2.5%
Köroğlu 2020	10	134.30	46.6000	15	265.80	184.5000	-0.87	[-1.71; -0.02]	1.6%
Leu 2019	703	103.70	65.7000	697	105.60	75.4000	-0.03	[-0.13; 0.08]	2.7%
Molina-Leyva 2018	14	116.30	55.7000	11	124.00	48.1000	-0.14	[-0.93; 0.65]	1.7%
Ruiz-Cabello 2016	54	88.70	66.9000	64	112.80	68.0000	-0.35	[-0.72; 0.01]	2.4%
Tuttolomondo 2015	180	122.60	101.5000	108	134.60	79.9000	-0.13	[-0.37; 0.11]	2.6%
Tuttolomondo 2020	179	116.00	56.6000	230	128.50	53.1000	-0.23	[-0.42; -0.03]	2.7%
Veissi 2016	87	160.10	99.3000	70	150.90	68.7000	0.11	[-0.21; 0.42]	2.5%
Viscogliosi 2013	18	95.50	50.7000	37	168.20	89.3000	-0.91	[-1.50; -0.32]	2.0%
Vitale 2018	705	146.70	71.0000	834	156.20	78.6000	-0.13	[-0.23; -0.03]	2.7%
Zupo 2020	265	105.80	62.5000	59	109.80	60.5000	-0.06	[-0.35; 0.22]	2.5%
Random effects model	3353			3731			-0.16	[-0.27; -0.06]	31.2%
Heterogeneity: $I^2 = 61\%$, $\tau^2 = 0.0183$, $p < 0.01$									
**Random effects model**	**21140**			**30058**			**-0.27**	**[-0.44; -0.11]**	**100.0%**
Heterogeneity: $I^2 = 95\%$, $\tau^2 = 0.2614$, $p < 0.01$									
Residual heterogeneity: $I^2 = 95\%$, $p < 0.01$									

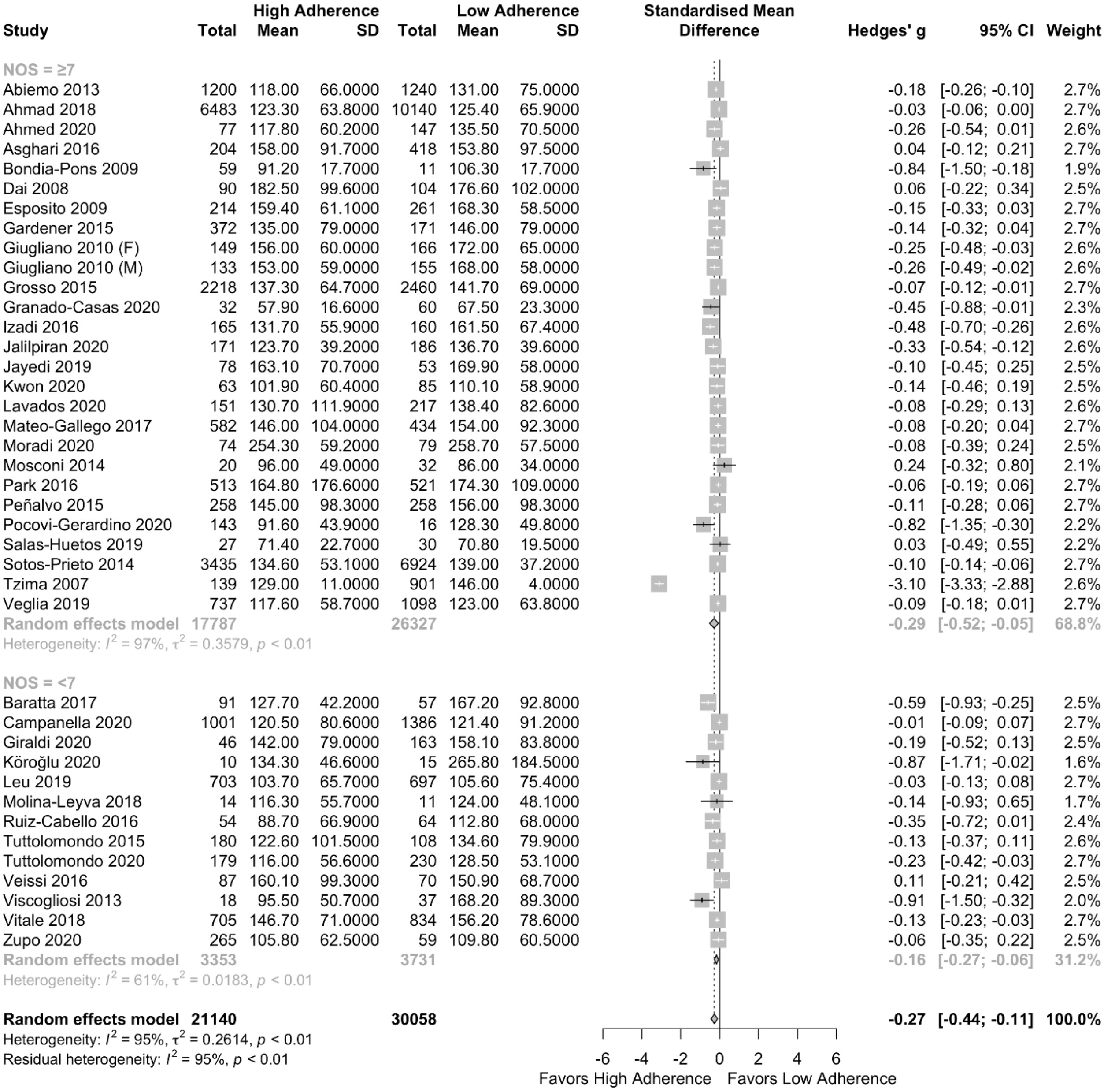

Supplementary Figure S4: Subgroup analysis based on the quality of studies regarding Triglycerides (mg/dL)
