# Supplementary material for: Impact of the Level of Adherence to Mediterranean Diet on the Parameters of Metabolic Syndrome: A Systematic Review and Meta-Analysis of Observational Studies
Source: Nutrients. 2021 Apr 30;13(5):1514. doi: 10.3390/nu13051514 (PMC8146502; doi:10.3390/nu13051514)
Supplement: Supplementary file 1 [file nutrients-13-01514-s001.zip › Supplementary Figure S5_FBG Supgroup.pdf]

Study	High Adherence Total	High Adherence Mean	High Adherence SD	Low Adherence Total	Low Adherence Mean	Low Adherence SD	Hedges' g	95% CI	Weight
NOS = ≥7									
Abiemo 2013	1200	89.40	8.8300	1240	89.90	8.9000	-0.06	[-0.14; 0.02]	3.2%
Ahmed 2020	77	93.50	15.8000	147	99.50	26.1000	-0.26	[-0.54; 0.02]	3.2%
Asghari 2016	204	95.50	27.4000	418	90.50	19.2000	0.22	[0.06; 0.39]	3.2%
Bondia-Pons 2009	59	93.60	14.4000	11	102.60	27.0000	-0.53	[-1.18; 0.12]	2.9%
Dai 2008	90	103.30	18.0000	104	98.20	18.4000	0.28	[0.00; 0.56]	3.2%
Esposito 2009	214	154.80	46.8000	261	153.00	39.6000	0.04	[-0.14; 0.22]	3.2%
Gardener 2015	372	108.00	52.0000	171	101.00	41.0000	0.14	[-0.04; 0.32]	3.2%
Giugliano 2010 (F)	149	157.00	34.0000	166	155.00	34.0000	0.06	[-0.16; 0.28]	3.2%
Giugliano 2010 (M)	133	154.00	34.0000	155	149.00	34.0000	0.15	[-0.09; 0.38]	3.2%
Grosso 2015	2218	96.10	25.6000	2460	96.70	25.4000	-0.02	[-0.08; 0.03]	3.2%
Izadi 2016	165	95.20	40.8000	160	118.80	54.4000	-0.49	[-0.71; -0.27]	3.2%
Jalilpiran 2020	171	104.70	18.3000	186	101.70	19.1000	0.16	[-0.05; 0.37]	3.2%
Jayedi 2019	78	160.40	54.4000	53	165.00	45.3000	-0.09	[-0.44; 0.26]	3.1%
Kwon 2020	63	97.40	19.0000	85	97.40	19.0000	0.00	[-0.33; 0.33]	3.1%
Mateo-Gallego 2017	582	98.90	16.4000	434	97.70	14.9000	0.08	[-0.05; 0.20]	3.2%
Moradi 2020	74	130.80	27.1000	79	129.10	26.1000	0.06	[-0.25; 0.38]	3.1%
Mosconi 2014	20	81.00	15.0000	32	76.00	9.0000	0.42	[-0.14; 0.99]	3.0%
Park 2016	513	101.30	18.8000	521	101.80	38.4000	-0.02	[-0.14; 0.11]	3.2%
Peñalvo 2015	258	99.90	18.2000	258	98.70	15.8000	0.07	[-0.10; 0.24]	3.2%
Salas-Huetos 2019	27	88.50	9.1000	30	88.00	9.3000	0.05	[-0.47; 0.57]	3.0%
Tzima 2007	139	92.00	2.0000	901	98.00	1.0000	-5.07	[-5.35; -4.79]	3.2%
Veglia 2019	737	97.70	20.7000	1098	112.70	32.8000	-0.52	[-0.62; -0.43]	3.2%
Random effects model	7543			8970			-0.24	[-0.70; 0.22]	69.5%
Heterogeneity: $I^2 = 98\%$, $\tau^2 = 1.2025$, $p < 0.01$									
NOS = <7									
Baratta 2017	91	100.20	16.6000	57	108.20	42.0000	-0.27	[-0.61; 0.06]	3.1%
Campanella 2020	1001	106.00	27.4000	1386	104.80	24.1000	0.05	[-0.03; 0.13]	3.2%
Giraldi 2020	46	99.90	43.4000	163	98.70	37.3000	0.03	[-0.30; 0.36]	3.1%
Köroğlu 2020	10	80.50	5.8000	15	96.40	19.8000	-0.97	[-1.82; -0.12]	2.7%
Leu 2019	703	98.90	22.9000	697	100.60	29.3000	-0.06	[-0.17; 0.04]	3.2%
Molina-Leyva 2018	14	93.40	22.4000	11	87.80	7.6000	0.31	[-0.49; 1.10]	2.7%
Ruiz-Cabello 2016	54	88.40	16.2000	64	85.60	16.8000	0.17	[-0.19; 0.53]	3.1%
Veissi 2016	87	162.40	82.7000	70	151.50	55.9000	0.15	[-0.16; 0.47]	3.1%
Viscogliosi 2013	18	93.00	9.3000	37	111.00	20.2000	-1.02	[-1.61; -0.42]	2.9%
Zupo 2020	265	89.80	12.8000	59	88.70	10.1000	0.09	[-0.19; 0.37]	3.2%
Random effects model	2289			2559			-0.04	[-0.16; 0.09]	30.5%
Heterogeneity: $I^2 = 63\%$, $\tau^2 = 0.0163$, $p < 0.01$									
**Random effects model**	**9832**			**11529**			**-0.21**	**[-0.54; 0.12]**	**100.0%**
Heterogeneity: $I^2 = 98\%$, $\tau^2 = 0.8724$, $p < 0.01$									
Residual heterogeneity: $I^2 = 98\%$, $p < 0.01$									

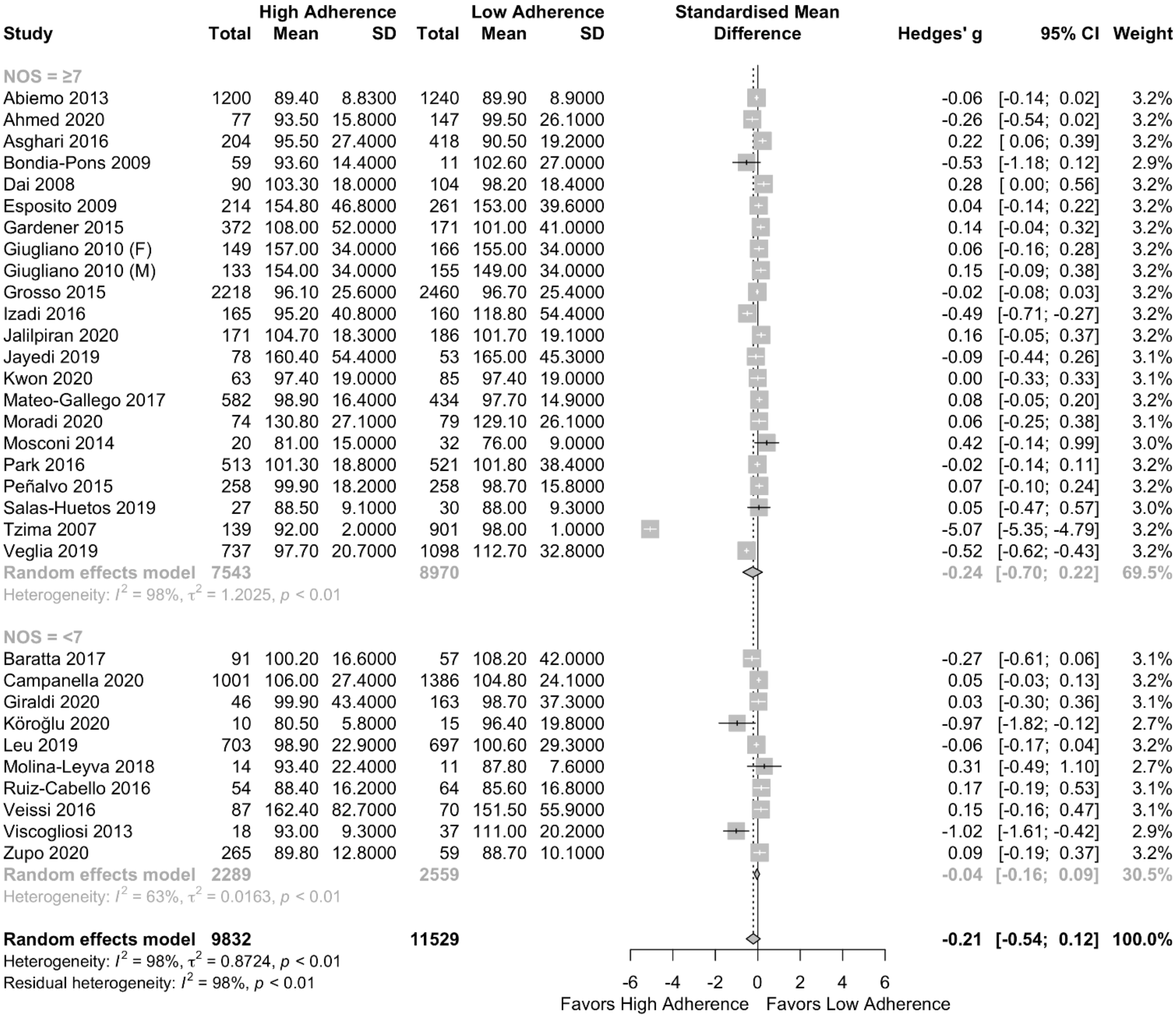

Supplementary Figure S5: Subgroup analysis based on the quality of studies regarding Fasting Blood Glucose (mg/dL)
