# Supplementary material for: Impact of the Level of Adherence to Mediterranean Diet on the Parameters of Metabolic Syndrome: A Systematic Review and Meta-Analysis of Observational Studies
Source: Nutrients. 2021 Apr 30;13(5):1514. doi: 10.3390/nu13051514 (PMC8146502; doi:10.3390/nu13051514)
Supplement: Supplementary file 1 [file nutrients-13-01514-s001.zip › Supplementary Figure S6_SBP Supgroup.pdf]

Study	High Adherence Total	High Adherence Mean	High Adherence SD	Low Adherence Total	Low Adherence Mean	Low Adherence SD	Hedges' g	95% CI	Weight
NOS = ≥7									
Abiemo 2013	1200	126.00	21.0000	1240	124.00	22.0000	0.09	[0.01; 0.17]	3.3%
Ahmad 2018	6483	121.50	7.4000	10140	125.00	14.8000	-0.28	[-0.31; -0.25]	3.3%
Ahmed 2020	77	130.40	25.7000	147	132.90	21.1000	-0.11	[-0.39; 0.17]	3.2%
Asghari 2016	204	114.20	16.9000	418	110.60	16.0000	0.22	[0.05; 0.39]	3.3%
Bondia-Pons 2009	59	116.30	10.9000	11	139.80	13.4000	-2.06	[-2.79; -1.32]	2.5%
Dai 2008	90	129.30	15.2000	104	130.30	16.3000	-0.06	[-0.35; 0.22]	3.2%
Gardener 2015	372	142.00	19.0000	171	145.00	22.0000	-0.15	[-0.33; 0.03]	3.3%
Grosso 2015	2218	136.90	20.8000	2460	138.70	20.9000	-0.09	[-0.14; -0.03]	3.3%
Izadi 2016	165	12.50	1.7000	160	12.50	1.8000	0.00	[-0.22; 0.22]	0.0%
Jalilpiran 2020	171	12.80	1.3000	186	13.00	1.4000	-0.15	[-0.36; 0.06]	0.0%
Jayedi 2019	78	132.00	114.5000	53	126.30	14.6000	0.06	[-0.29; 0.41]	3.1%
Kwon 2020	63	119.20	12.1000	85	116.90	12.7000	0.18	[-0.14; 0.51]	3.1%
Lavados 2020	151	148.00	25.0000	217	151.00	26.2000	-0.12	[-0.32; 0.09]	3.3%
Mateo-Gallego 2017	582	125.00	14.2000	434	125.00	14.5000	0.00	[-0.12; 0.12]	3.3%
Moradi 2020	74	104.20	21.2000	79	105.70	20.4000	-0.07	[-0.39; 0.25]	3.1%
Mosconi 2014	20	122.00	12.0000	32	117.00	15.0000	0.35	[-0.21; 0.92]	2.8%
Park 2016	513	129.90	21.5000	521	125.90	14.2000	0.22	[0.10; 0.34]	3.3%
Peñalvo 2015	258	126.00	14.6000	258	125.00	14.0000	0.07	[-0.10; 0.24]	3.3%
Pocovi-Gerardino 2020	143	118.70	19.4000	16	115.40	14.9000	0.17	[-0.34; 0.69]	2.8%
Salas-Huetos 2019	27	127.00	11.0000	30	126.30	11.4000	0.06	[-0.46; 0.58]	2.8%
Tzima 2007	139	125.00	1.0000	901	128.00	1.0000	-3.00	[-3.22; -2.78]	3.2%
Veglia 2019	737	135.00	18.0000	1098	147.00	18.0000	-0.67	[-0.76; -0.57]	3.3%
Random effects model	13824			18761			-0.25	[-0.60; 0.10]	63.0%
Heterogeneity: $I^2 = 98\%$, $\tau^2 = 0.6142$, $p < 0.01$									
NOS = <7									
Baratta 2017	91	130.00	15.1000	57	130.00	15.2000	0.00	[-0.33; 0.33]	3.1%
Campanella 2020	1001	127.90	17.1000	1386	120.90	16.5000	0.42	[0.34; 0.50]	3.3%
Hu 2013	3956	148.40	19.1000	3349	148.90	19.0000	-0.03	[-0.07; 0.02]	3.3%
Köroğlu 2020	10	115.80	6.5000	15	120.00	17.2000	-0.29	[-1.09; 0.52]	2.3%
Leu 2019	703	119.70	18.1000	697	117.50	17.3000	0.12	[0.02; 0.23]	3.3%
Molina-Leyva 2018	14	135.40	18.3000	11	127.50	8.3000	0.52	[-0.29; 1.32]	2.3%
Ruiz-Cabello 2016	54	121.10	16.2000	64	123.40	16.0000	-0.14	[-0.50; 0.22]	3.1%
Tuttolomondo 2015	180	138.60	24.7000	108	143.10	27.6000	-0.17	[-0.41; 0.07]	3.2%
Tuttolomondo 2020	179	132.60	20.5000	230	133.90	18.3000	-0.07	[-0.26; 0.13]	3.3%
Veissi 2016	87	129.20	18.3000	70	131.70	19.8000	-0.13	[-0.45; 0.18]	3.1%
Vitale 2018	705	133.30	23.7000	834	135.30	14.9000	-0.10	[-0.20; 0.00]	3.3%
Zupo 2020	265	128.60	16.1000	59	129.20	18.1000	-0.04	[-0.32; 0.25]	3.2%
Random effects model	7245			6880			0.01	[-0.11; 0.14]	37.0%
Heterogeneity: $I^2 = 90\%$, $\tau^2 = 0.0305$, $p < 0.01$									
**Random effects model**	**21069**			**25641**			**-0.15**	**[-0.38; 0.07]**	**100.0%**
Heterogeneity: $I^2 = 97\%$, $\tau^2 = 0.3933$, $p < 0.01$									
Residual heterogeneity: $I^2 = 97\%$, $p < 0.01$									

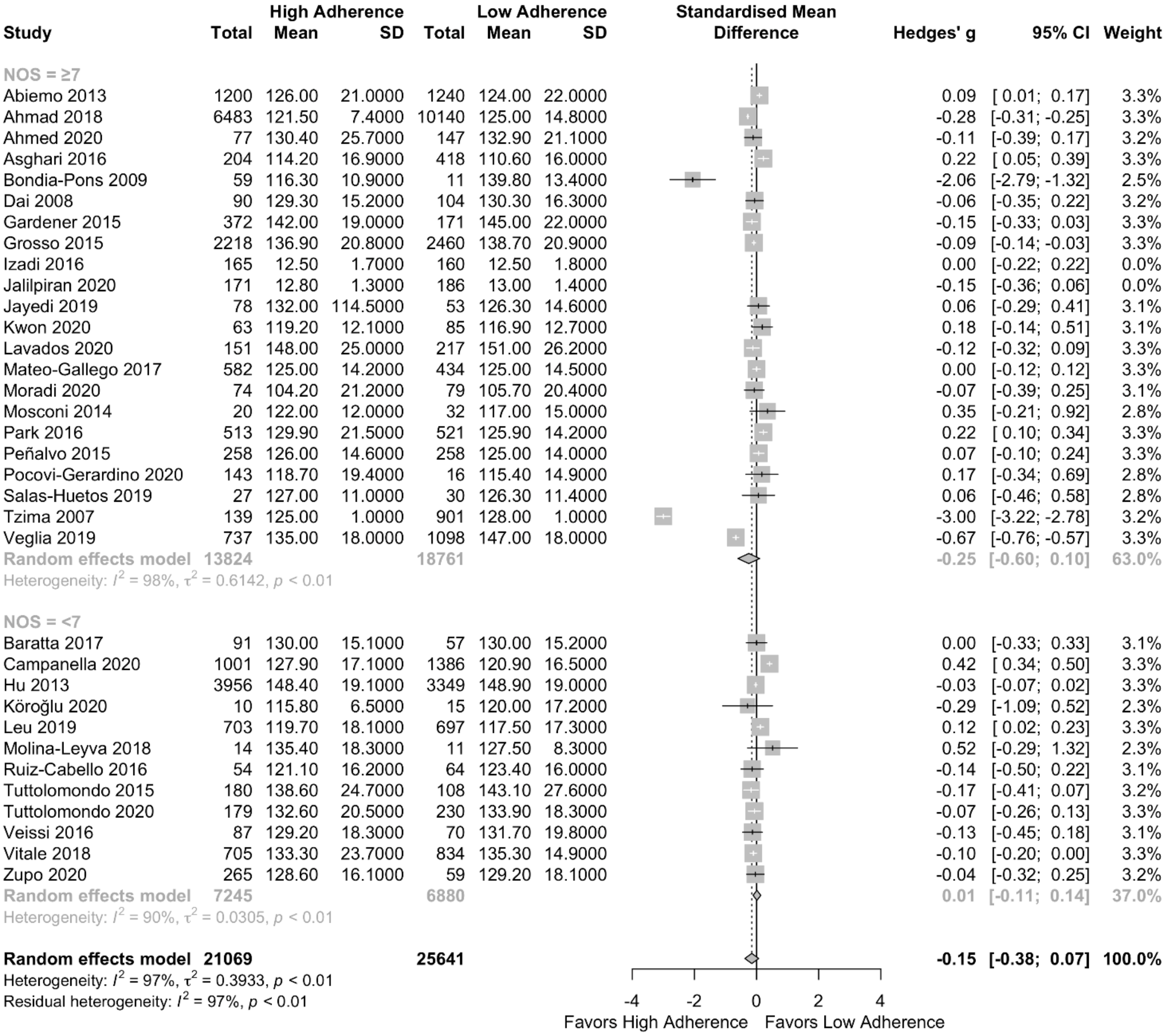

Supplementary Figure S6: Subgroup analysis based on the quality of studies regarding Systolic Blood Pressure (mmHg)
