# Supplementary material for: Impact of the Level of Adherence to Mediterranean Diet on the Parameters of Metabolic Syndrome: A Systematic Review and Meta-Analysis of Observational Studies
Source: Nutrients. 2021 Apr 30;13(5):1514. doi: 10.3390/nu13051514 (PMC8146502; doi:10.3390/nu13051514)
Supplement: Supplementary file 1 [file nutrients-13-01514-s001.zip › Supplementary File S1.pdf]

Quality assessment of the present meta-analysis according to MOOSE checklist for observational studies (From: Stroup DF, Berlin JA, Morton SC, et al, for the Meta-analysis Of Observational Studies in Epidemiology (MOOSE) Group. Meta-analysis of Observational Studies in Epidemiology. A Proposal for Reporting. JAMA. 2000;283(15):2008-2012. doi: 10.1001/jama.283.15.2008.)

Item No	Recommendation	Reported on Page No
**Reporting of background should include**		
1	Problem definition	03
2	Hypothesis statement	03
3	Description of study outcome(s)	03
4	Type of exposure or intervention used	03
5	Type of study designs used	04
6	Study population	04
**Reporting of search strategy should include**		
7	Qualifications of searchers (e.g., librarians and investigators)	04
8	Search strategy, including time period included in the synthesis and key words	04 + Suppl. File 2
9	Effort to include all available studies, including contact with authors	04
10	Databases and registries searched	04
11	Search software used, name and version, including special features used (e.g., explosion)	04
12	Use of hand searching (e.g., reference lists of obtained articles)	04
13	List of citations located and those excluded, including justification	05 + Figure 1
14	Method of addressing articles published in languages other than English	04
15	Method of handling abstracts and unpublished studies	04
16	Description of any contact with authors	NA
**Reporting of methods should include**		
17	Description of relevance or appropriateness of studies assembled for assessing the hypothesis to be tested	04
18	Rationale for the selection and coding of data (e.g., sound clinical principles or convenience)	04
19	Documentation of how data were classified and coded (e.g., multiple raters, blinding and interrater reliability)	NA
20	Assessment of confounding (e.g., comparability of cases and controls in studies where appropriate)	04-05
21	Assessment of study quality, including blinding of quality assessors, stratification or regression on possible predictors of study results	04-05
22	Assessment of heterogeneity	05
23	Description of statistical methods (e.g., complete description of fixed or random effects models, justification of whether the chosen models account for predictors of study results, dose-response models, or cumulative meta-analysis) in sufficient detail to be replicated	05
24	Provision of appropriate tables and graphics	Figures 1,2,3,4,5,6 Tables 1,2,3, Supplementary File 3, Suppl. Figures 1a-e,2,3,4,5,6
**Reporting of results should include**		
25	Graphic summarizing individual study estimates and overall estimate	NA

26	Table giving descriptive information for each study included	Tables 1,2
27	Results of sensitivity testing (e.g., subgroup analysis)	17-22 + Suppl Figures 2,3,4,5,6
28	Indication of statistical uncertainty of findings	24-25
**Reporting of discussion should include**		
29	Quantitative assessment of bias (e.g., publication bias)	08
30	Justification for exclusion (e.g., exclusion of non-English language citations)	04, 25
31	Assessment of quality of included studies	05-06
**Reporting of conclusions should include**		
32	Consideration of alternative explanations for observed results	23-24
33	Generalization of the conclusions (i.e., appropriate for the data presented and within the domain of the literature review)	24
34	Guidelines for future research	25
35	Disclosure of funding source	26
