# Supplementary material for: Impact of the Level of Adherence to Mediterranean Diet on the Parameters of Metabolic Syndrome: A Systematic Review and Meta-Analysis of Observational Studies
Source: Nutrients. 2021 Apr 30;13(5):1514. doi: 10.3390/nu13051514 (PMC8146502; doi:10.3390/nu13051514)
Supplement: Supplementary file 1 [file nutrients-13-01514-s001.zip › Supplementary File S2.pdf]

Supplementary File 2: Search terms of electronic databases

Pubmed (11/01/2021)

(https://www.ncbi.nlm.nih.gov/pubmed/)

Search: Mediterranean diet AND Adherence Sort by: Publication Date

("diet, mediterranean"[MeSH Terms] OR ("diet"[All Fields] AND "mediterranean"[All Fields]) OR "mediterranean diet"[All Fields] OR ("mediterranean"[All Fields] AND "diet"[All Fields])) AND ("adherance"[All Fields] OR "adhere"[All Fields] OR "adhered"[All Fields] OR "adherence"[All Fields] OR "adherences"[All Fields] OR "adherent"[All Fields] OR "adherents"[All Fields] OR "adherer"[All Fields] OR "adherers"[All Fields] OR "adheres"[All Fields] OR "adhering"[All Fields])

Translations

Mediterranean diet: "diet, mediterranean"[MeSH Terms] OR ("diet"[All Fields] AND "mediterranean"[All Fields]) OR "mediterranean diet"[All Fields] OR ("mediterranean"[All Fields] AND "diet"[All Fields])

Adherence: "adherance"[All Fields] OR "adhere"[All Fields] OR "adhered"[All Fields] OR "adherence"[All Fields] OR "adherences"[All Fields] OR "adherent"[All Fields] OR "adherents"[All Fields] OR "adherer"[All Fields] OR "adherers"[All Fields] OR "adheres"[All Fields] OR "adhering"[All Fields]

Results: 2203

Scopus (11/01/2021-based on TIT-ABST-KEYWORDS)

(https://www.scopus.com/home.uri)

TIT-ABST-KEYWORDS: ("Mediterranean diet" AND Adherence)

Results: 2309

Web of Science (11/01/2021-based on ALL FIELDS)

(www.webofknowledge.com)

("Mediterranean diet" AND Adherence)

Results: 2940

Embase (11/01/2021- based on ALL FIELDS)

(https://www.embase.com/)

(Mediterranean diet and adherence).mp. [mp=title, abstract, heading word, drug trade name, original title, device manufacturer, drug manufacturer, device trade name, keyword, floating subheading word, candidate term word]

Results: 3005

CENTRAL (11/01/2021- based on ALL FIELDS)

(https://www.cochranelibrary.com/)

("Mediterranean diet" AND Adherence)

Results: 496

Google Scholar (11/01/2021- based on TIT & exclude patents and citations)

(https://scholar.google.gr/schhp?hl=el)

("Mediterranean diet" AND Adherence)

Records identified through reference searching: 2

Total number of studies: N= 2720
