# Supplementary material for: Impact of the Level of Adherence to Mediterranean Diet on the Parameters of Metabolic Syndrome: A Systematic Review and Meta-Analysis of Observational Studies
Source: Nutrients. 2021 Apr 30;13(5):1514. doi: 10.3390/nu13051514 (PMC8146502; doi:10.3390/nu13051514)
Supplement: Supplementary file 1 [file nutrients-13-01514-s001.zip › Supplementary File S3.pdf]

Supplementary File 3: Quality assessment according to the New Castle Ottawa Scale (NOS)

Study ID	Representativeness of the sample	Sample size	Non-respondents	Ascertainment of the exposure (risk factor)	Comparability of subjects in different outcome groups on the basis of design or analysis. Confounding factors controlled.	Assessment of outcome	Statistical test	TOTAL STARS	NOS
Abiemo et al 2013	*		*	*	**	**	*	8	Good
Ahmad et al 2018		*	*	*	**	**	*	8	Good
Ahmed et al 2020			*	*	**	**	*	7	Good
Alvarez-Leon et al 2006	*			*	**	*	*	6	Satisfactory
Aridi et al 2020	*		*	*	**	**	*	8	Good
Asghari et al 2016	*		*	*	**	**	*	8	Good
Baratta et al 2017			*	*		**	*	5	Satisfactory
Barnaba et al 2020			*	*		**	*	5	Satisfactory
Bondia-Pons et al 2009	*	*	*	*		**	*	7	Good
Campanella et al 2020	*	*		*		**	*	6	Satisfactory
Dai et al 2008	*	*	*	*	**	**	*	9	Very good
Esposito et al 2009			*	*	**	**	*	7	Good
Gardener et al 2015	*	*	*	*	**	**	*	9	Very good

Giraldi et al 2020			*		**		*	4	Unsatisfactory
Giugliano et al 2010 (M)		*	*	*	**	**	*	8	Good
Giugliano et al 2010 (W)		*	*	*	**	**	*	8	Good
Granado-Casas et al 2020	*		*	*	**	**	*	8	Good
Grosso et al 2015	*	*	*	*	**	**	*	9	Very good
Hu et al 2013		*	*	*		**	*	6	Satisfactory
Huang et al 2013	*		*	*	**	**	*	8	Good
Izadi et al 2016			*	*	**	**	*	7	Good
Jalipiran et al 2020	*	*	*	*	**	**	*	9	Very good
Jayedi et al 2019		*	*	*	**	**	*	8	Good
Karayiannis et al 2017				*	**	**	*	6	Satisfactory
Kesse-Guyot et al 2013	*	*	*	*	**	**	*	9	Very good
Kim et al 2018	*	*	*	*	**	**	*	9	Very good
Köroğlu & Adıgüzel 2020			*	*		**		4	Unsatisfactory
Kwon et al 2020			*	*	**	**	*	7	Good
Lavados et al 2020			*	*	**	**	*	7	Good
Leu et al 2019	*		*	*		**	*	6	Satisfactory

Mahdavi-Roshan et al 2017			*	*		**	*	5	Satisfactory
Mateo-Gallego 2017			*	*	**	**	*	7	Satisfactory
Mattei et al 2017	*			*	**	**	*	7	Good
Mayr et al 2019	*	*	*	*	**	**	*	9	Very good
Mirmiran et al 2015	*		*	*	**	**	*	8	Good
Molina-Leyva et al 2019			*	*				2	Unsatisfactory
Moradi et al 2020		*	*	*	**	**	*	8	Good
Mosconi et al 2014			*	*	**	**	*	7	Good
Mziwira et al 2015				*	**	**	*	6	Satisfactory
Park et al 2016	*		*	*	**	**	*	8	Good
Peñalvo et al 2015		*	*	*	**	**	*	8	Good
Pocovi-Gerardino et al 2020			*	*	**	**	*	7	Good
Roldan et al 2019	*			*		**	*	5	Satisfactory
Ruiz-Cabello et al 2016			*	*		**	*	5	Satisfactory
Rumawas et al 2009			*	*	**	**	*	7	Good
Salsa-Huetos et al 2019			*	*	**	**	*	7	Good

Sotos-Prieto et al 2014		*	*	*	**	**	*	8	Good
Steffen et al 2014	*		*	*	**	**	*	8	Good
Tortosa et al 2007			*		**			3	Unsatisfactory
Tuttolomondo et al 2015		*	*	*		**	*	6	Satisfactory
Tuttolomondo et al 2020		*	*	*		**	*	6	Satisfactory
Tzima et al 2007	*	*	*	*	**	**	*	9	Very good
Veglia et al 2019		*	*	*	**	**	*	8	Good
Veissi et al 2016			*	*		**	*	5	Satisfactory
Viscogliosi et al 2013			*	*		**	*	5	Satisfactory
Vitale et al 2018			*	*		**	*	5	Satisfactory
Yang et al 2014				*		**	*	4	Unsatisfactory
Zupo et al 2020			*	*		**	*	5	Satisfactory
